# Transcript splicing optimizes the thymic self-antigen repertoire to suppress autoimmunity

**DOI:** 10.1172/JCI179612

**Published:** 2024-10-15

**Authors:** Ryunosuke Muro, Takeshi Nitta, Sachiko Nitta, Masayuki Tsukasaki, Tatsuo Asano, Kenta Nakano, Tadashi Okamura, Tomoki Nakashima, Kazuo Okamoto, Hiroshi Takayanagi

**Affiliations:** 1 Department of Immunology, Graduate School of Medicine and Faculty of Medicine, The University of Tokyo, Tokyo, Japan.; 2 Division of Molecular Pathology, Research Institute for Biomedical Sciences, Tokyo University of Science, Chiba, Japan.; 3 Department of Osteoimmunology, Graduate School of Medicine and Faculty of Medicine, The University of Tokyo, Tokyo, Japan.; 4 Department of Laboratory Animal Medicine, Research Institute, National Center for Global Health and Medicine, Tokyo, Japan.; 5 Department of Cell Signaling, Graduate School of Medical and Dental Sciences, Tokyo Medical and Dental University, Tokyo, Japan.; 6 Division of Immune Environment Dynamics, Cancer Research Institute, Kanazawa University, Kanazawa, Japan.

**Keywords:** Immunology, T cells, Tolerance

## Abstract

Immunological self-tolerance is established in the thymus by the expression of virtually all self-antigens, including tissue-restricted antigens (TRAs) and cell-type–restricted antigens (CRAs). Despite a wealth of knowledge about the transcriptional regulation of TRA genes, posttranscriptional regulation remains poorly understood. Here, we show that protein arginine methylation plays an essential role in central immune tolerance by maximizing the self-antigen repertoire in medullary thymic epithelial cells (mTECs). Protein arginine methyltransferase-5 (Prmt5) was required for pre-mRNA splicing of certain key genes in tolerance induction, including *Aire* as well as various genes encoding TRAs. Mice lacking Prmt5 specifically in thymic epithelial cells exhibited an altered thymic T cell selection, leading to the breakdown of immune tolerance accompanied by both autoimmune responses and enhanced antitumor immunity. Thus, arginine methylation and transcript splicing are essential for establishing immune tolerance and may serve as a therapeutic target in autoimmune diseases as well as cancer immunotherapy.

## Abstract 2

Protein arginine methylation controls splicing of self-antigen transcripts expressed in thymic epithelial cells to suppress autoimmunity.

## Graphical abstract

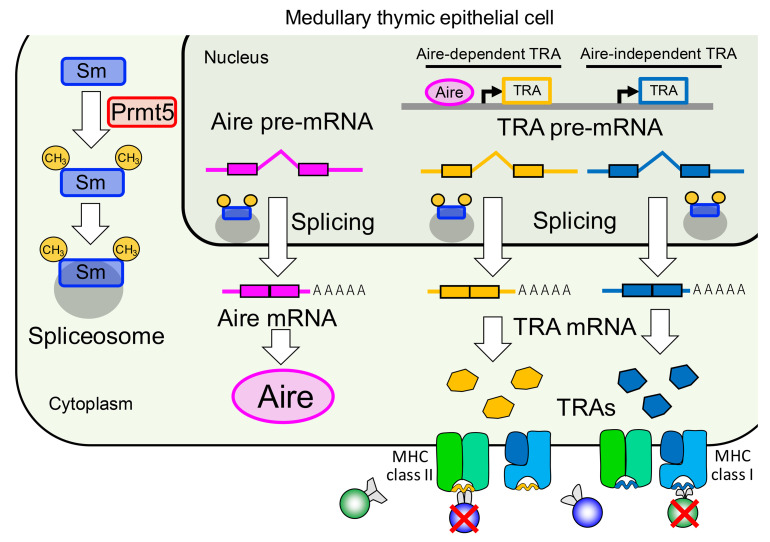

## Introduction

The thymus is the primary lymphoid organ that provides specialized microenvironments for generating the T cell repertoire (1). In 2 anatomically discrete regions within the thymus, the cortex and the medulla, functionally distinct thymic epithelial cells (TECs) play key roles in the stepwise selection of developing T cells (thymocytes). Cortical TECs (cTECs) support the positive selection of CD4^+^CD8^+^ double-positive (DP) thymocytes into CD4^+^ single-positive (CD4SP) or CD8SP thymocytes. Positively selected CD4SP and CD8SP thymocytes migrate to the medulla, where a diverse array of self-antigens are displayed to eliminate self-reactive SP thymocytes by negative selection or induce agonistic selection of Tregs. Medullary TECs (mTECs) are the most well characterized cells that produce various tissue-restricted antigens (TRAs) in the medulla, contributing to the generation of a self-tolerant T cell receptor (TCR) repertoire and, thereby, the establishment of central immune tolerance (2, 3).

TRA expression in mTECs, termed “promiscuous gene expression,” is mediated by complex and finely regulated transcriptional machinery. The best-studied factor that controls the transcription of TRA genes is autoimmune regulator (Aire), a nuclear protein expressed in mTECs (4). Aire promotes the transcription of a number of genes including TRAs via multiple processes: activation of superenhancers (5, 6), modulation of chromatin accessibility (7, 8), and transcriptional elongation (9, 10). Mice, rats, and humans with dysfunctional Aire protein develop multiorgan autoimmunity, indicating the physiological significance of Aire-dependent TRA expression in immune tolerance (11–13). Aire regulates approximately 30%–40% of the TRAs expressed in mTECs (2), while the other TRAs (Aire-independent TRAs) are thought to be regulated by other transcriptional factors, such as Fezf2 and SpiB (14). The regulatory function of Aire and Fezf2 is under the control of the chromatin remodeler CHD4 (15). Such diverse TRA expression is enabled partly by the mTEC heterogeneity. A fraction of mTECs undergo differentiation into atypical epithelial cells such as thymic tuft cells, the development of which depends on the transcriptional regulators Pou2f3, Hipk2, and Sox4 (16–19). These terminally differentiated mTECs including thymic tuft cells, keratinocyte-like mTECs, and microfold cell-like mTECs express specific TRAs for each epithelial cell lineage, which may contribute to maximization of the variety of self-antigens available for T cell selection. A recent study illustrated that thymic fibroblasts, a nonepithelial stromal cell, contribute to the suppression of autoimmunity by acting as a valid source of fibroblast antigens, which represent cell-type–restricted antigens (CRAs) (20). Thus, multiple layers of cellular and molecular mechanisms, although not yet fully understood, underlie the generation of the diverse self-antigen repertoire in the thymic medulla.

Arginine methylation, the addition of methyl group(s) onto the arginine residues in proteins, plays a key role in various eukaryotic processes such as epigenetic regulation, DNA repair, protein synthesis, signal transduction, and mRNA processing (21). Nine protein arginine methyltransferases (PRMTs) have been identified in mammals and are categorized into 3 types on the basis of their catalytic activities: type I PRMTs (PRMT1, PRMT2, PRMT3, CARM1, PRMT6, and PRMT8) produce asymmetric dimethylated arginine; type II PRMTs (PRMT5 and PRMT9) produce symmetric dimethylated arginine; and type III PRMT (PRMT7) generates monomethylated arginine. These PRMTs target both nuclear and cytoplasmic proteins, including histones, small nuclear ribonucleoproteins, transcriptional regulators, and signaling proteins (22). Yet despite the versatile and critically important role of PRMTs in diverse cellular functions, their role in mTECs has not to our knowledge been investigated.

Among the PRMT members, PRMT5 is a symmetric dimethyltransferase that has recently attracted attention as a regulator of key cellular processes and potential therapeutic target for cancer (23). Since systemic deletion of the *Prmt5* gene results in early embryonic lethality in mice, its physiological and pathological roles have been investigated using cell-type–specific or inducible gene deletion in mice. A series of studies have demonstrated that Prmt5 is required for the development and/or maintenance of various cell types such as germ cells, neuronal cells, and hematopoietic cells (21). We and others have reported that Prmt5 controls the development and function of lymphocytes, including T cells and B cells (24–27). Thus, Prmt5 plays important roles in a variety of immune cells, although its function in the stromal cells that support immune cell development remains totally unclear.

In this study, we explored the significance of arginine methylation in the gene expression that occurs in mTECs. Prmt5 was identified as the major PRMT expressed in mTECs, and mice lacking Prmt5 specifically in TECs exhibited an impaired development of mTECs, but not cTECs. Transcriptome analyses revealed that Prmt5 is required for the pre-mRNA splicing of Aire and various other genes, including both Aire-dependent and Aire-independent TRAs. TEC-specific, Prmt5-deficient mice developed a spontaneous autoimmune phenotype with autoantibody production and lymphocyte infiltration into peripheral organs, while they were also protected from experimental metastasis. This suggests that the Prmt5-dependent pre-mRNA splicing in mTECs controls the balance between immune tolerance and tumor immunity. Thus, our results highlight the arginine methylation that generates the self-antigen repertoire required for inducing T cell tolerance in the thymus.

## Results

### Prmt5 is the major PRMT expressed in mTECs.

To gain insight into the significance of arginine methylation in TECs, we explored public databases to examine the mRNA expression of the PRMT family of genes in various thymic cells. Prmt5 was found to be most highly expressed in TECs, especially in functionally mature mTECs (mTEC^hi^), rather than in DP, CD4SP, or CD8SP thymocytes (Supplemental Figure 1A; supplemental material available online with this article; https://doi.org/10.1172/JCI179612DS1). We analyzed the mTEC transcriptome data from a lineage-tracing mouse model (17), which made it possible to distinguish the mTEC differentiation stages from each other according to the expression of Aire and MHC-II (Supplemental Figure 1B). Prmt5 was expressed at the highest level in the early stage of Aire-expressing mature mTECs, and the expression level of Prmt5 during mTEC differentiation clearly correlated with that of Aire. The expression of Prmt9, another type II PRMT, was much lower than that of Prmt5 in the mTEC subsets. Thus, Prmt5 is the major symmetric arginine dimethyltransferase in mature mTECs, suggesting a role in the differentiation and/or function of mTECs.

### Impaired mTEC development in TEC-specific, Prmt5-deficient mice.

**Figure 1:**
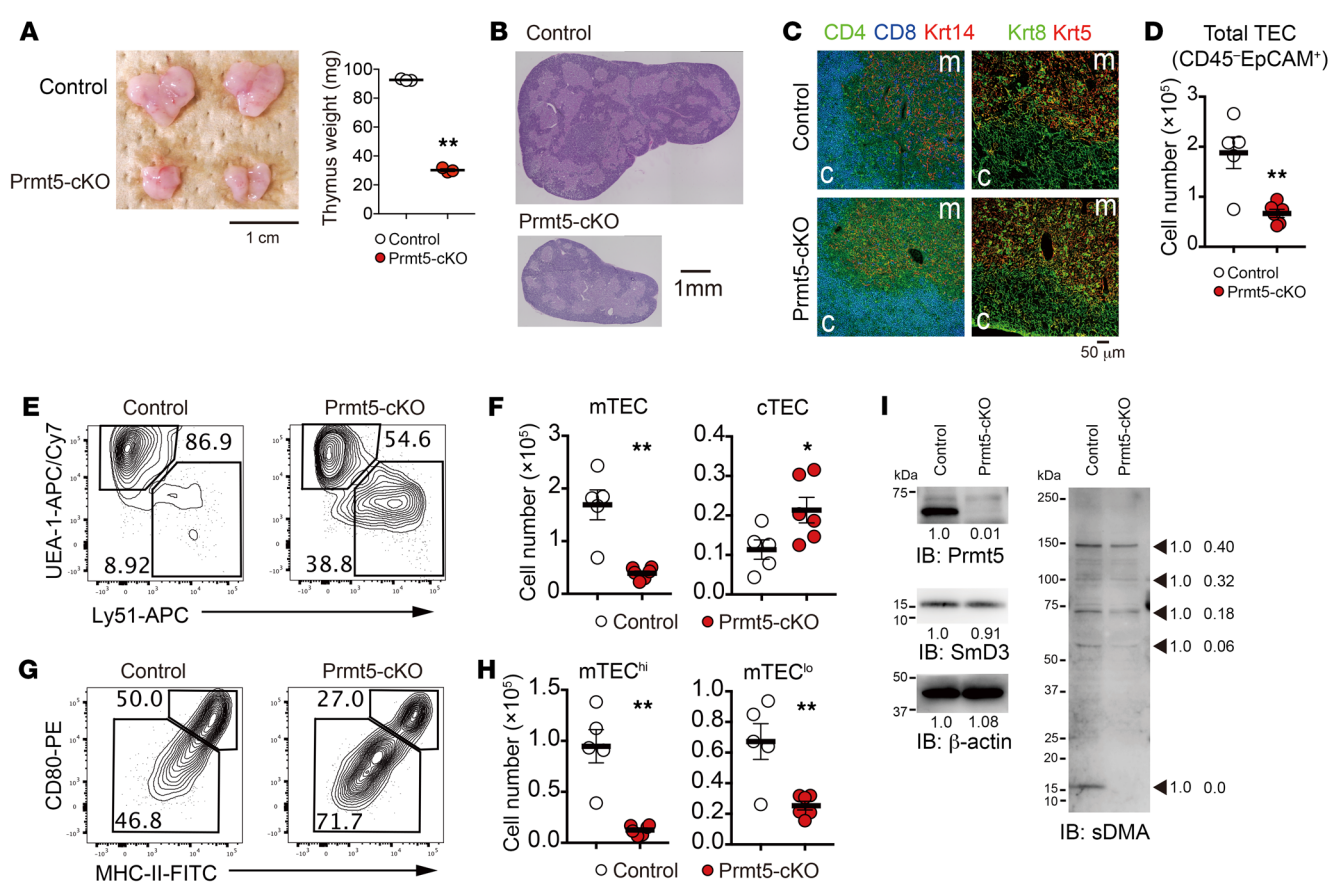
TEC-specific Prmt5 deficiency impairs mTEC development. (**A**) Representative photograph of thymi from 4-week-old control (Prmt5^fl/fl^) and Prmt5-cKO (Prmt5^fl/fl^ Foxn1-Cre) mice and thymic weight. Scale bar: 1 cm. (**B** and **C**) Thymic sections from 4-week-old mice were stained with H&E (**B**) or CD4 (green), CD8 (blue), keratin 14 (Krt14, red), keratin 8 (Krt8, green, and keratin 5 (Krt5, red). (**C**). The data shown represent 2 independent experiments. Scale bars: 1 mm (**B**) and 50 μm (**C**). c, cortex; m, medulla. (**D**) Number of CD45^–^EpCAM^+^ TECs (cells per thymus lobe) from 4- to 5-week-old control mice (*n* = 5) and Prmt5-cKO mice (*n* = 6). Each circle indicates 1 mouse. (**E** and **F**) Flow cytometric analysis of UEA-1 and Ly51 expression on gated TECs (CD45^–^EpCAM^+^) from the indicated mice (**E**). Graphs depict the number of cTECs (Ly51^+^) and mTECs (UEA-1^+^) (**F**). (**G** and **H**) Flow cytometric analysis of CD80 and MHC-II expression on gated mTECs (CD45EpCAM^+^UEA-1^+^) (**G**). Graphs depict the number of CD80^hi^MHC-II^hi^ mTECs (mTEC^hi^ cells) and CD80^lo^MHC-II^lo^ mTECs (mTEC^lo^ cells) (**H**). (**I**) Isolated total mTECs (EpCAM^+^CD45^–^UEA-1^+^Ly51^–^) from control mice and Prmt5-cKO mice were subjected to SDS-PAGE (1.0 × 10^5^ cell equivalents per lane) followed by immunoblotting with antibodies against Prmt5, SmD3, symmetric dimethyl-arginine (sDMA), and β-actin. Two independent experiments were performed. The arrows indicate the proteins with symmetrically dimethylated arginines. Numbers below the bands and beside the arrow indicate the relative intensity of each band, measured with ImageJ. *P < 0.5 and **P < 0.01, by 2-tailed Student’s *t* test (**A**, **D**, **F**, and **H**).

To investigate the role of Prmt5 in TECs, we crossed Prmt5^fl/fl^ mice with Foxn1-Cre mice (24, 28). The Foxn1-Cre Prmt5^fl/fl^ (hereafter referred to as Prmt5-cKO mice) had a normal body size and displayed a normal skin phenotype but had a smaller thymus with a significantly lower number of thymocytes and a lighter thymus weight than did Prmt5^fl/fl^ (control) mice (Figure 1A and Supplemental Figure 2A). There was no apparent difference in the frequency of DN, DP, or CD4SP cells between the control and Prmt5-cKO mice, whereas the frequency of CD8SP cells and Tregs was slightly (but significantly) reduced in the Prmt5-cKO mice (Supplemental Figure 2, B–D). As the number of total thymocytes was markedly reduced (Supplemental Figure 2A), the number of each T-lineage thymocyte population was reduced in Prmt5-cKO mice (Supplemental Figure 2, C and D).

Histological analysis revealed that Prmt5-cKO mice had normal compartmentalization of the thymic cortex (in which DP cells localize) and medulla (in which CD4SP and CD8SP cells localize) (Figure 1B). The expression of keratin 8 (the cTEC maker) and keratin 5 (the mTEC marker) was also normally detectable, indicating that the distribution of cTECs and mTECs was not altered in the Prmt5-cKO mice (Figure 1C). Flow cytometric analysis of Liberase-digested thymic cells revealed a significant reduction of EpCAM^+^CD45^–^ TECs in the Prmt5-cKO mice compared with control mice (Figure 1D). Among the TEC populations, UEA-1^+^Ly51^–^ mTECs were severely reduced, while UEA-1^–^Ly51^+^ cTECs were slightly increased (Figure 1, E and F). In particular, the CD80^hi^MHC-II^hi^ mTECs (mTEC^hi^) that contain an Aire-expressing mature cell population were most severely influenced by Prmt5 deficiency (an 87% reduction) (Figure 1, G and H). The CD80^lo^MHC-II^lo^ mTECs (mTEC^lo^) that comprise immature mTECs and terminally differentiated mTECs were also reduced in Prmt5-cKO mice (62% reduction). We found no difference in the frequency of Ki67^+^ proliferating cells or 7-amino-actinomycin D^+^ (7AAD^+^) dead cells (Supplemental Figure 2, E–H). These results indicate that Prmt5 was required for the development of mTECs.

Western blot analysis confirmed the deletion of PRMT5 protein expression in mTECs derived from Prmt5-cKO mice (Figure 1I). Prmt5-deficient mTECs exhibited reduced protein levels with symmetrically dimethylated arginines, and, of particular note, a 15 kDa band, which corresponds to the SmD3 protein that is known to be a major target of symmetric arginine dimethylation by Prmt5 (29, 30), was substantially diminished (Figure 1I). These results indicate that Prmt5 functioned as an active symmetric arginine dimethyltransferase that targeted SmD3 in mTECs.

### Prmt5 controls the splicing of Aire pre-mRNA.

**Figure 2:**
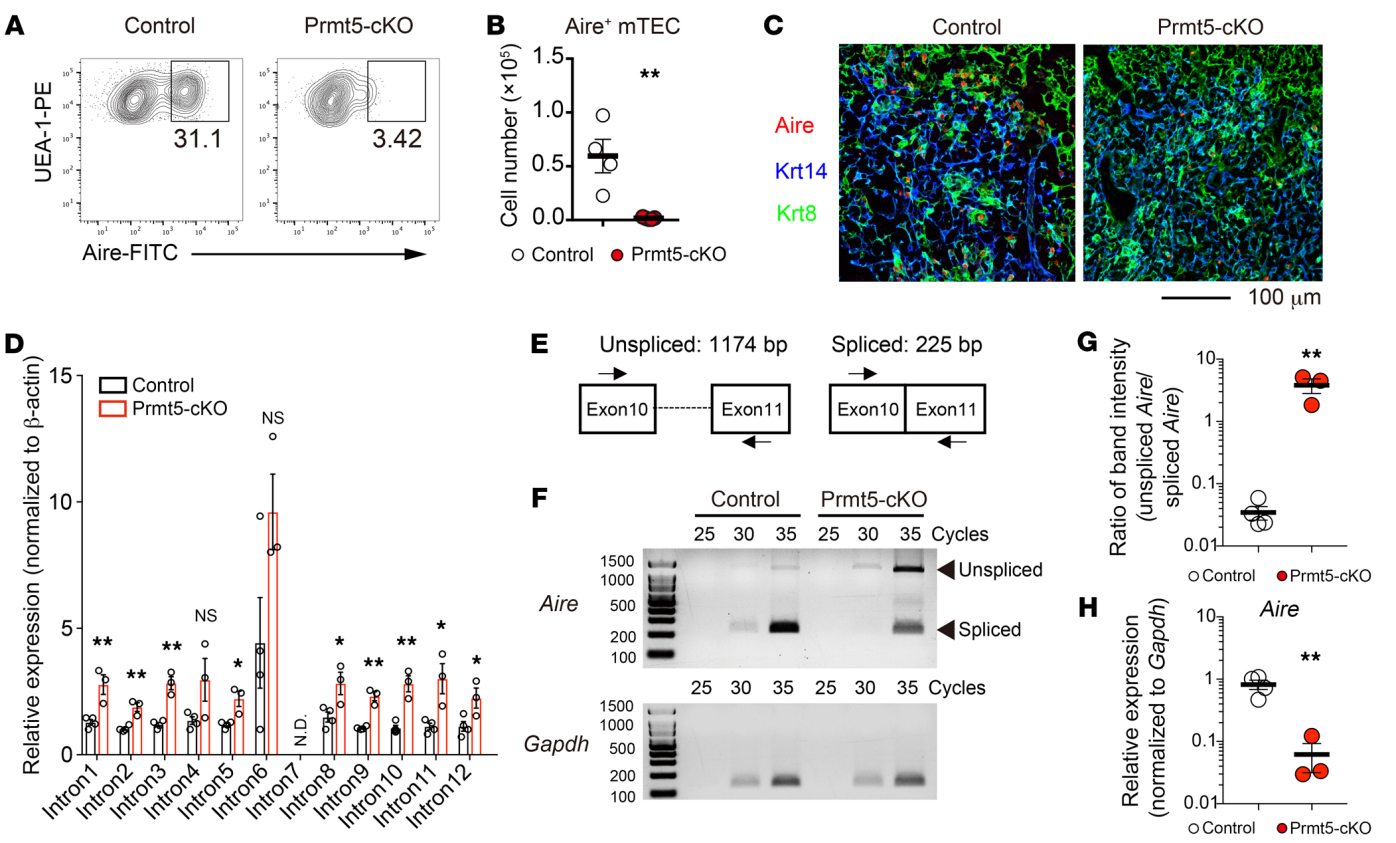
Prmt5 is required for Aire expression. (**A** and **B**) Flow cytometric analysis of Aire-expressing mTECs (CD45^–^EpCAM^+^UEA-1^+^) from 4- to 5-week-old control mice (Prmt5^fl/fl^, *n* = 4) and Prmt5-cKO mice (Prmt5^fl/fl^ Foxn1-Cre, *n* = 5) (**A**). The graph indicates the number of Aire^+^ mTECs (cells per thymus lobe) (**B**). Each circle indicates one mouse. (**C**) Immunohistochemical detection of Aire-expressing cells in the indicated mice. Thymic sections were stained for Aire (red), Krt14 (an mTEC marker, shown in blue), and Krt8 (a cTEC marker: green). The data are representative of 2 independent experiments. Scale bar: 100 μm. (**D**) Intronic retention of Aire mRNA in Prmt5-deficient mTEC^hi^ cells. The relative expression of the indicated intron regions in sorted mTEC^hi^ cells from control mice (*n* = 4) and Prmt5-cKO (*n* = 3) was determined by qRT-PCR. N.D., not detected. (**E**) Schematic of unspliced and spliced Aire mRNA. The primers used in **F** are indicated with arrows. (**F**–**H**) Semiquantitative RT-PCR analysis of unspliced and spliced Aire mRNA in Prmt5-deficient mTEC^hi^ cells. The cDNA samples synthesized from RNA isolated from control mTEC^hi^ cells (*n* = 4) and Prmt5-deficient mTEC^hi^ cells (*n* = 3) were amplified with a set of primers as shown in **E** and analyzed by agarose gel electrophoresis (**F**). *Gapdh* served as an internal control. The graph shows the ratio of band intensity for unspliced (1,174 bp) versus spliced (225 bp) *Aire* mRNA (**G**), and for spliced (225 bp) *Aire* mRNA versus *Gapdh* (**H**). Band intensity was measured with ImageJ. *P < 0.5 and **P < 0.01, by 2-tailed Student’s *t* test (**B**, **D**, **G**, and **H**).

We analyzed Aire-expressing mTECs in Prmt5-cKO mice to study the effect of Prmt5 deficiency on mTEC function. The frequency and number of Aire-expressing mTECs detected by flow cytometry were severely reduced in Prmt5-cKO mice (Figure 2, A and B). Immunohistochemical analysis confirmed a drastic reduction of Aire expression in the medulla of the thymus (Figure 2C).

Given that Prmt5 deficiency impairs the arginine methylation of the Sm proteins that are required for optimum pre-mRNA splicing (21–23), we assessed the splicing status of *Aire* mRNA in Prmt5-deficient mTEC^hi^ cells. Quantitative reverse transcription PCR (qRT-PCR) analysis revealed that the Aire transcripts in Prmt5-deficient mTEC^hi^ cells contained more intron sequences than did those in control mTEC^hi^ cells (Figure 2D). Semiquantitative RT-PCR analysis with a set of primers spanning exons 10 and 11 showed that the unspliced form of Aire pre-mRNA containing the retained intron 10 was markedly increased and that the amount of spliced Aire mRNA decreased in Prmt5-deficient mTEC^hi^ cells (Figure 2, E–H, and Supplemental Figure 3), suggesting that mature Aire mRNA expression was substantially reduced in Prmt5-deficient mTEC^hi^ cells. Thus, Prmt5-mediated arginine dimethylation was required for pre-mRNA splicing of the *Aire* gene to generate protein-coding mRNA.

### Prmt5 is essential for the expression of Aire-dependent and -independent TRAs.

**Figure 3:**
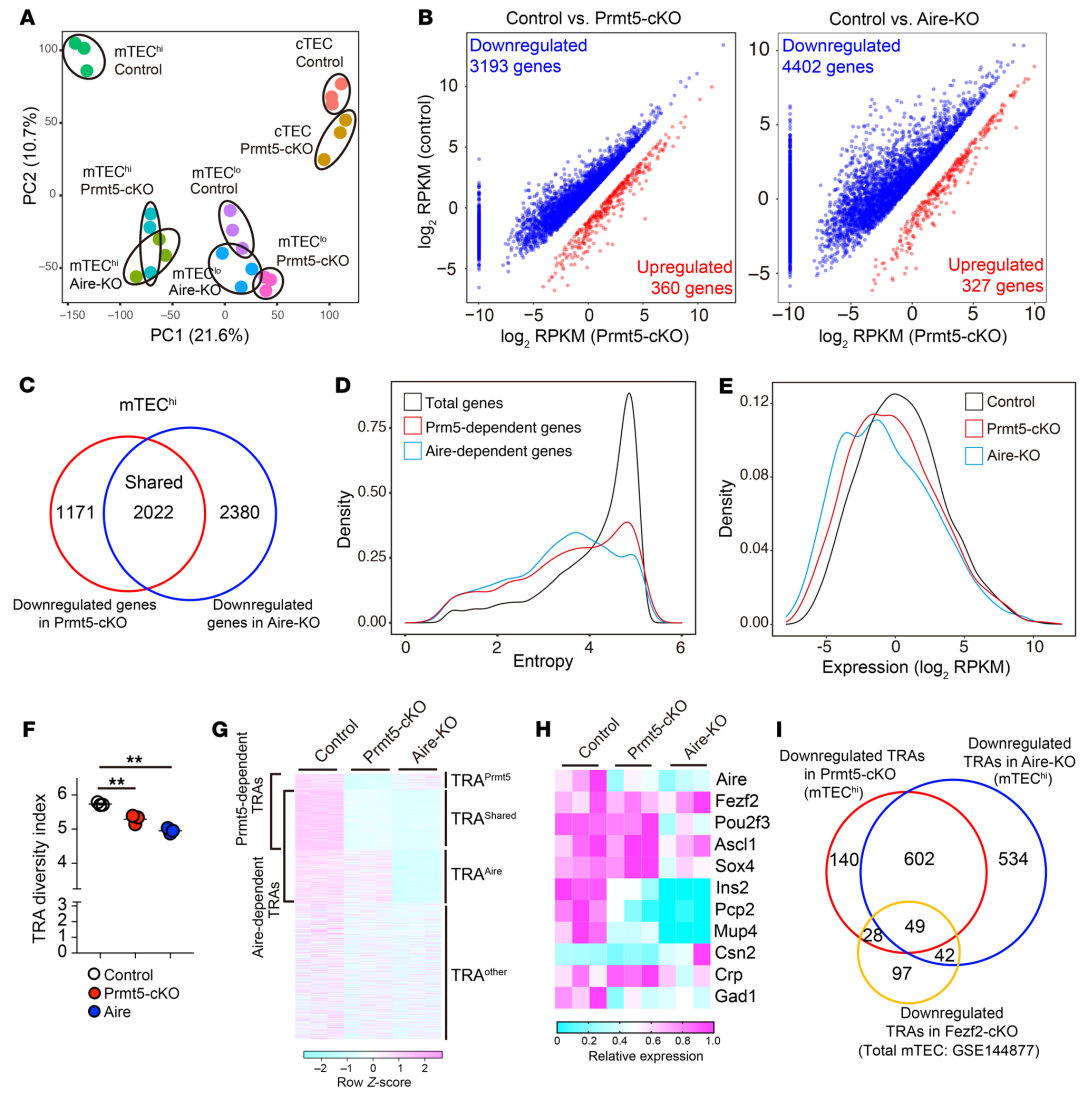
Transcriptome analysis of Prmt5-deficient mTECs. (**A**) PCA of RNA-Seq data. The indicated TEC subsets isolated from control mice (Prmt5^fl/fl^, *n* = 3), Prmt5-cKO mice (Prmt5^fl/fl^ Foxn1-Cre, *n* = 3), and Aire-KO mice (*n* = 3) were subjected to RNA-Seq analysis. The percentages on each axis indicate the variance contribution. (**B**) Scatter plots of differentially expressed genes (*P* < 0.05, and fold change >2 [red] or <0.5 [blue]) in Prmt5-deficient or Aire-deficient mTEC^hi^ cells. Data represent the mean of log_2_(reads per kilobase of exon per million mapped reads [RPKM]). Significance was determined using the unpaired, 2-tailed Student’s t test. (**C**) Venn diagram showing the overlap between the set of genes significantly downregulated in Prmt5-deficient mTEC^hi^ cells and Aire-deficient mTEC^hi^ cells. (**D**) Density plot showing the entropy score of individual genes expressed in mTEC^hi^ cells. The total genes expressed in mTEC^hi^ cells (mean of RPKM >0 in control mTEC^hi^ cells, 19,324 genes: black line), genes downregulated (*P* < 0.05, fold change <0.5) in Prmt5-deficient mTEC^hi^ cells (2,652 genes: red line), and genes downregulated (*P* < 0.05, fold change <0.5) in Aire-deficient mTEC^hi^ cells (3,677 genes: blue line). (**E**) Density plot showing gene expression of total TRAs (entropy score <3.0) in mTEC^hi^ cells from control mice (black), Prmt5-cKO mice (red), and Aire-KO mice (blue). (**F**) The diversity index of the TRAs (entropy score <3.0) expressed in mTEC^hi^ cells was evaluated using the Shannon-Weaver model. **P < 0.01, by 1-way ANOVA followed by Dunnett’s multiple-comparison test. (**G**) Expression heatmap of total TRA genes in mTEC^hi^ cells (entropy score <3.0). (**H**) Relative expression of representative transcriptional factors and TRAs in mTEC^hi^ cells shown as a heatmap. (**I**) Venn diagram showing the overlap between a set of TRA genes under the control of Prmt5, Aire, and Fezf2 (GSE144877).

We performed RNA-Seq analysis on sorted mTEC^hi^ and mTEC^lo^ cells as well as cTECs from control and Prmt5-cKO mice. Since Aire expression was markedly reduced in Prmt5-deficient mTECs, mTEC^hi^ and mTEC^lo^ cells from Aire-deficient mice were analyzed for comparison. The Prmt5-deficient mTEC^hi^ cells were completely different from the control mTEC^hi^ cells but similar to the Aire-deficient mTEC^hi^ cells in the gene expression pattern upon principal component analysis (PCA) (Figure 3A). Gene expression in cTECs was not markedly different between the control and Prmt5-cKO mice. Approximately 3,000 genes were significantly (*P* < 0.05, fold change <0.5) downregulated in Prmt5-deficient mTEC^hi^ cells, whereas more than 4,000 genes were downregulated in Aire-deficient mTEC^hi^ cells (Figure 3B). Approximately 2,000 genes were downregulated in both groups in common, indicating that Prmt5 regulates gene expression in mTECs partly through Aire (Figure 3C).

To define TRAs objectively, we applied a mathematical method to evaluate the tissue specificity of gene expression. We identified 3,322 genes as TRA genes (entropy score <3.0) in a mouse gene expression catalog (GSE10246) (Supplemental Figure 4, A and B) (31). TRAs comprise 30% and 33% of the Prmt5- and Aire-dependent genes, respectively (Figure 3D). Density distribution analysis revealed that the overall expression of TRAs in mTEC^hi^ cells was decreased in Prmt5-cKO and Aire-KO mice (Figure 3E). We analyzed the RNA-Seq data using the Shannon-Weaver diversity index in order to evaluate the variety of TRAs expressed in mTEC^hi^ cells. The TRA diversity index was significantly lower in the Prmt5-cKO group and Aire-KO group than in the control group, indicating that Prmt5 enhances the diversity of TRAs expressed in mTEC^hi^ cells (Figure 3F). The 2,866 TRA genes expressed in mTEC^hi^ were categorized into 4 groups: TRA^Prmt5^ (168 genes [5.9%], downregulated only in Prmt5-cKO mice); TRA^Aire^ (576 genes [20.1%], downregulated only in Aire-KO mice); TRA^shared^ (651 genes [22.7%], downregulated in both Prmt5-cKO and Aire-KO mice); TRA^other^ (1,471 genes, [51.3%]) (Figure 3G and Supplemental Figure 4C). These results indicate that Prmt5 controls not only Aire-dependent but also Aire-independent TRA expression, suggesting the regulatory mechanism linked to the intrinsic function of Prmt5. It is likely that the induction of TRA^Aire^ expression in Prmt5-deficient mTEC^hi^ cells was dependent on a low but functional level of Aire protein.

Certain transcription factors have previously been suggested to control Aire-independent TRA expression (14). However, our RNA-Seq data demonstrated that known mTEC-related transcription factors such as *Fezf2*, *Pou2f3*, *Ascl1*, and *Sox4* were normally expressed in Prmt5-deficient mTEC^hi^ cells (Figure 3H). Thus, it is unlikely that Prmt5-dependent TRAs were induced through these transcription factors. Indeed, only a fraction of the TRAs downregulated in Prmt5-deficient mTEC^hi^ cells (77 genes) overlapped with Fezf2-dependent TRAs, while as many as 651 Prmt5-dependent TRAs (79.5%) were dependent on Aire (Figure 3I).

### Prmt5 is required for the pre-mRNA splicing of TRA genes.

**Figure 4:**
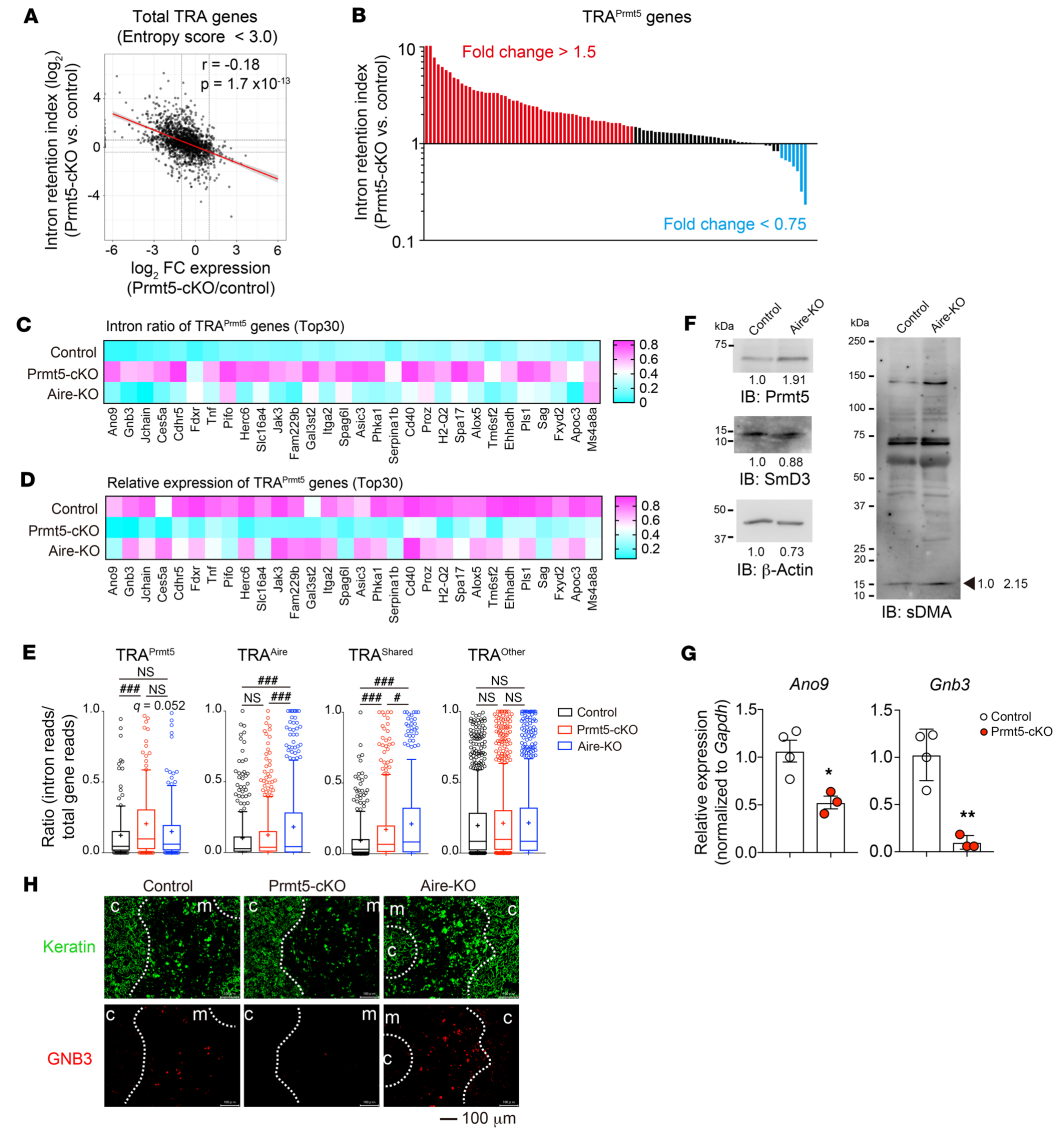
Prmt5 promotes the mRNA processing of TRA genes. (**A**) Scatter plot of the intron retention index versus the fold change of gene expression (Prmt5-cKO/control) for total TRA genes expressed in mTEC^hi^ cells. Pearson’s correlation coefficient (*r*) and *P* values are shown. (**B**) Intron retention index for Prmt5-induced TRA genes (total read count ≥10 in Prmt5-deficient mTEC^hi^ cells). Each bar indicates an individual Prmt5-induced TRA gene. Genes with increased (fold change >1.5) or decreased (fold change <0.75) intron retention in Prmt5-deficient mTEC^hi^ cells are shown in red and blue, respectively. (**C** and **D**) Heatmaps showing the intron ratio (**C**) and relative mRNA expression (**D**) of Prmt5-induced TRA genes (top 30 in **B**) in mTEC^hi^ cells from the indicated mice. The data indicate the mean value for the intron ratio and relative RPKM. (**E**) Graphs show the mean ratio of intron retention of the TRA genes categorized in Figure 3G (total read count >0 in each group). Significance was determined using the FDR of the Benjamini and Hochberg method (^#^*q* < 0.05 and ^###^*q* < 0.001). + indicates the mean. (**F**) Total mTECs (EpCAM^+^CD45^–^UEA-1^+^Ly51^–^) isolated from control or Aire-KO mice were subjected to SDS-PAGE followed by immunoblotting with antibodies against Prmt5, SmD3, sDMA, and β-actin. Representative data from 2 independent experiments are shown. Numbers below the bands and beside the arrow indicate the relative intensity of each band. (**G**) Relative expression levels of *Ano9* and *Gnb3* in sorted mTEC^hi^ cells were analyzed by qRT-PCR (control, *n* = 4; Prmt5-cKO, *n* = 3). *P < 0.5 and **P < 0.01, by unpaired, 2-tailed Student’s *t* test. (**H**) Thymic sections from 4- to 5-week-old control mice, Prmt5-cKO mice, and Aire-KO mice were stained with antibodies against pan-keratin (green) and GNB3 (red). Representative images from 2 independent experiments are shown. Scale bar: 100 μm.

Given the role of Prmt5 in pre-mRNA splicing, we explored the effect of Prmt5 deficiency on the generation of mature mRNAs encoding TRAs. We examined the ratio of intron reads to total reads for individual genes (intron retention index) in the RNA-Seq data. We found that the decrease in TRA expression in Prmt5-cKO mTEC^hi^ cells significantly correlated with the increase in intron retention (Figure 4A). Approximately half of the TRA^Prmt5^ genes showed a 1.5-fold or greater increase in the intron retention index in Prmt5-cKO mTEC^hi^ cells (Figure 4B). The representatives (top 30) of the TRA^Prmt5^ showed an increased intron ratio (intron reads per total reads) in Prmt5-deficient mTEC^hi^ cells but not in Aire-deficient mTEC^hi^ cells (Figure 4C). The ratio of intron retention was inversely correlated with the relative mRNA expression level of each TRA (Figure 4D). The Prmt5 deficiency significantly increased the intron ratio of TRA^Prmt5^ as well as that of TRA^shared^, but not that of TRA^Aire^ (Figure 4E). Reciprocally, the deficiency of Aire caused an increase in the intron retention of TRA^Aire^ and TRA^share^, but not that of TRA^Prmt5^. These results indicate that Prmt5-mediated pre-mRNA splicing was required for the optimum expression of a set of TRA genes. Aire was also important for the splicing of a different set of TRA genes (Figure 4E), consistent with a previous report showing that Aire interacts with spliceosome factors to promote pre-mRNA splicing (32, 33). Aire-deficient mTECs exhibited normal expression of Prmt5 and normal symmetric arginine dimethylation of the SmD3 protein (Figure 4F), indicating that Aire and Prmt5 control pre-mRNA splicing via different molecular mechanisms.

Furthermore, we tested whether Prmt5 deficiency affects individual TRA expression at the mRNA and protein levels. qRT-PCR analysis confirmed that the amount of spliced mRNA of *Ano9* and *Gnb3*, representative of TRA^Prmt5^, was significantly reduced in Prmt5-deficient mTEC^hi^ cells (Figure 4G), whereas intron retention levels were significantly increased (Supplemental Figure 5A). Gel electrophoresis of RT-PCR products with a set of primers spanning exon 12 to exon 14 of Ano9 showed a complete loss of spliced mature *Ano9* mRNA but an increased of intron-containing *Ano9* pre-mRNAs in Prmt5-deficient mTEC^hi^ cells (Supplemental Figure 5, B–E). Immunohistochemical analysis revealed GNB3 protein expression in a fraction of mTECs in control and Aire-deficient mice but was nearly undetectable in Prmt5-cKO mice (Figure 4H). These results demonstrate that Prmt5 promotes pre-mRNA splicing of transcripts encoding TRAs, which is required for the efficient production of self-antigens in mTECs.

### Prmt5 controls Aire-independent gene expression for TRA production and mTEC differentiation.

**Figure 5:**
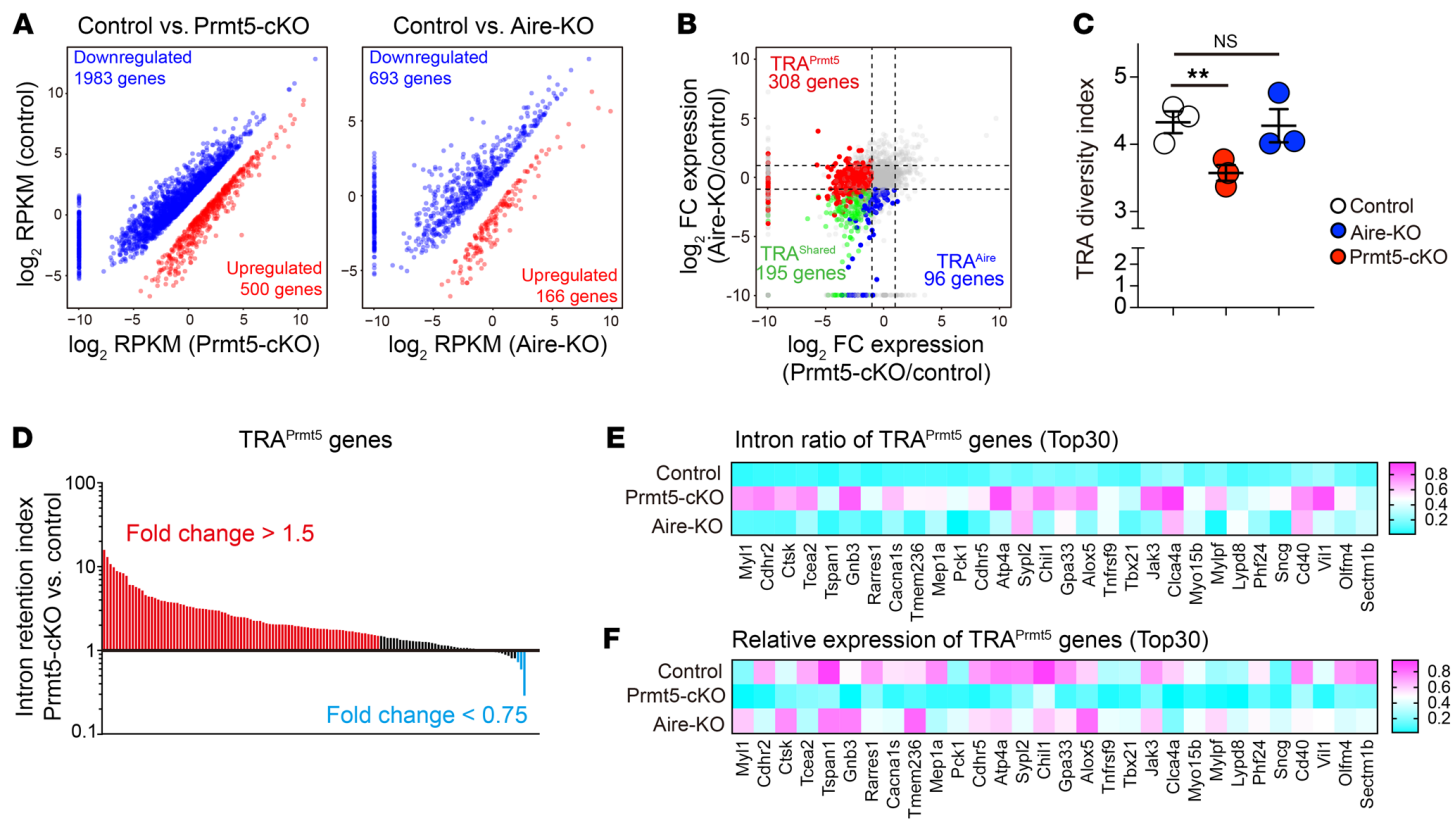
Prmt5 controls Aire-independent gene expression in mTEC^lo^ cells. (**A**) Scatter plots of differentially expressed genes (*P* < 0.05, and fold change >2 [red] or <0.5 [blue]) in Prmt5-deficient or Aire-deficient mTEC^lo^ cells. Data represent the mean of log_2_ (RPKM). Significance was determined using the unpaired, 2-tailed Student’s t test. (**B**) Scatter plot comparing the TRA gene expression ratio (log_2_) between Prmt5-cKO/control and Aire-KO/control. (**C**) The diversity index of TRA genes (entropy score <3.0) expressed in mTEC^lo^ cells was evaluated using the Shannon-Weaver model. **P < 0.01, by 1-way ANOVA followed by Dunnett’s multiple-comparison test. (**D**) Intron retention index for Prmt5-induced TRA genes (read count ≥10 in Prmt5-deficient mTEC^lo^ cells). Each bar indicates individual Prmt5-induced TRA genes. Genes with increased (fold change >1.5) or decreased (fold change <0.75) intron retention in Prmt5-deficient mTEC^lo^ cells are shown in red and blue, respectively. (**E** and **F**) Heatmaps showing the intron ratio (**E**) and relative mRNA expression (**F**) of TRA^Prmt5^ genes (top 30 in **D**) in mTEC^lo^ cells from the indicated mice. Data indicate the mean value for the intron ratio and relative RPKM.

Recent single-cell transcriptome analyses of mouse and human thymus have demonstrated that the mTEC^lo^ cell compartment is markedly heterogeneous, containing immature mTECs and terminally differentiated mTECs that express distinct sets of TRAs (14, 19). RNA-Seq analysis revealed that the number of downregulated genes was 1,983 and 693 in Prmt5-cKO and Aire-KO mTEC^lo^ cells, respectively (Figure 5A), suggesting that Prmt5 controls a higher number of genes in mTEC^lo^ cells than Aire. Among the TRAs expressed in mTEC^lo^ cells, the number of TRA^Prmt5^ genes (308 TRAs) was higher than that of TRA^Aire^ genes (96 TRAs) (Figure 5B). The Shannon-Weaver diversity index indicated that the Prmt5-cKO mTEC^lo^ cells had reduced diversity of TRA expression, whereas Aire-KO mTEC^lo^ cells did not (Figure 5C). These results suggest that Prmt5, rather than Aire, controls the diversity of self-antigens expressed in mTEC^lo^ cells. As in the case of mTEC^hi^ cells, TRAs^Prmt5^ in mTEC^lo^ cells displayed an increase in the intron retention index (Figure 5D), as demonstrated by an inverse correlation between the intron retention ratio and relative mRNA expression (Figure 5, E and F). These results indicate that Prmt5 controls the pre-mRNA splicing of the TRA genes expressed in the mTEC^lo^ cell population.

The mTEC^lo^ cells contained thymic tuft cells, a recently discovered cell subset that features a gene expression pattern and cell morphology similar to that of peripheral tuft cells (16, 17). The RNA-Seq data showed that the expression of thymic tuft cell–associated genes was markedly reduced in Prmt5-deficient mTEC^lo^ cells (Supplemental Figure 6A). Flow cytometric as well as histological analysis revealed that Dclk1^+^ thymic tuft cells were significantly reduced in Prmt5-cKO mice (Supplemental Figure 6, B–D). Thus, Prmt5 promoted thymic tuft cell generation, suggesting an essential role for Prmt5-mediated pre-mRNA splicing in the differentiation program of a certain subset of mTECs.

The mTEC^lo^ cells also contained mTEC progenitor cells, which give rise to Aire-expressing mature mTECs in response to the stimulation of receptor activator for NF-κB (RANK) (2, 3, 14). In addition to RANK signal, the development of mTECs was cooperatively controlled by CD40 and lymphotoxin β receptor (LTβR). Our RNA-Seq data showed that the expression of RANK and CD40 was significantly downregulated, with increased intron retention, whereas the expression of LTβR (or its intron retention) was not altered (Supplemental Figure 6, E and F). These results suggest that impaired development of mTECs in Prmt5-cKO mice was caused by inhibition of pre-mRNA splicing of RANK and CD40.

### Prmt5-cKO mice exhibit autoimmunity and enhanced antitumor immunity.

**Figure 6:**
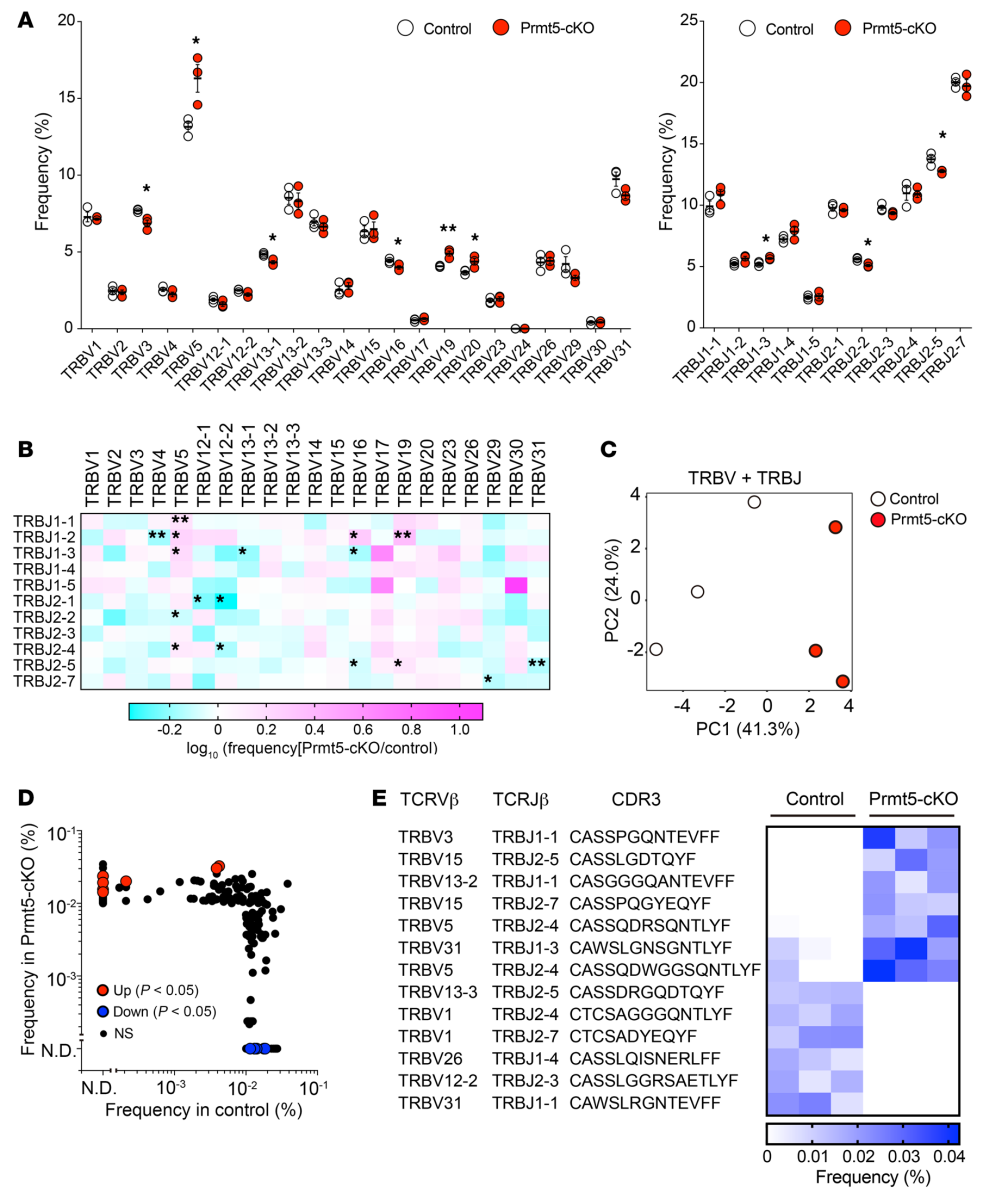
Altered T cell selection in Prmt5-cKO mice. (**A**) Frequency of TCRβ V and J region usage in mature CD4SP cells (CD4^+^CD8^–^TCRβ^+^CD25^–^CD69^lo^CD62L^hi^) from control (Prmt5^fl/fl^, *n* = 3) and Prmt5-cKO (Prmt5^fl/fl^ Foxn1-Cre, *n* = 3) mice. (**B**) Heatmap showing the ratio of the detection frequency of TCRβ V-J chain pairing usage (Prmt5-cKO/control). (**C**) PCA of TCRβ V and J chain data. (**D**) Scatter plot of filtered TCRβ reads (detection frequency >0.01%) comparing control and Prmt5-cKO mice. The TCRβ chains with significantly changed detection frequency (*P* < 0.05) are shown in red or blue. (**E**) Heatmap indicates the frequency (percentage) of the TCRβ chain sequences in individual mice. *P < 0.5; significance was determined by unpaired, 2-tailed Student’s *t* test (**A**, **B**, **D**, and **E**).

To explore the effect of Prmt5 deficiency on thymic T cell selection, we performed a high-throughput deep sequencing analysis of the TCR-Vβ chain expressed on mature CD4SP cells (CD4^+^CD8TCRβ^+^CD25^–^CD69^lo^CD62L^hi^) (Supplemental Figure 7, A and B) (34). The usage of the TCR-Vβ, -Jβ, and Vβ-Jβ combinations was significantly different between the control and Prmt5-cKO mice (Figure 6, A and B). PCA analysis using TCR-Vβ and -Jβ detection frequency data revealed that the TCR repertoire formed in the thymus of Prmt5-cKO mice is distinct from that of control mice (Figure 6C). We sorted common and frequent TCRs (detection frequency >0.01 %) and found a significant increase in the detection frequency of 7 TCRs in Prmt5-cKO mice compared with control mice (Figure 6, D and E). We further investigated the repertoire of TCR-Vβ in thymic CD8SP cells and splenic CD8^+^ T cells by flow cytometry and found that the usage of certain TCR-Vβ was significantly different in Prmt5-cKO mice (Supplemental Figure 7C). These results suggest that TEC-specific Prmt5 deficiency affects the selection of both thymic CD4SP and CD8SP cells and permits the development of certain T cells that are deleted in the normal thymus.

**Figure 7:**
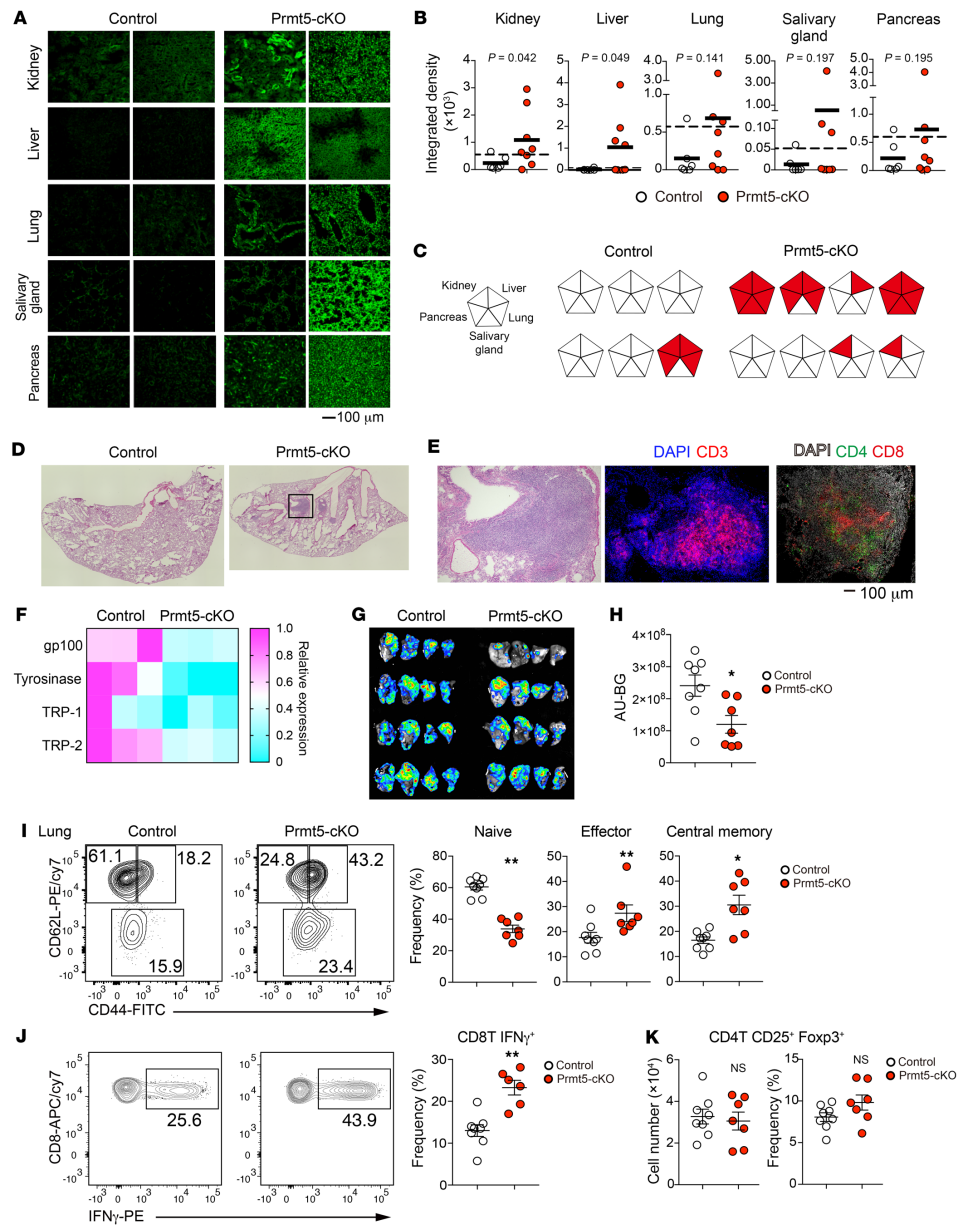
Loss of Prmt5 in TECs causes autoimmunity but reinforces antitumor immunity. (**A**) Frozen sections of the indicated organs from Rag2-deficient mice were stained with serum from 7- to 12-month-old control mice (Prmt5^fl/fl^, *n* = 6) and Prmt5-cKO mice (Prmt5^fl/fl^ Foxn1-Cre, *n* = 8). Scale bar: 100 μm. (**B**) Summary of the integrated density of the fluorescence signals shown in **A**. Dashed lines indicate the average of control integrated density values plus 2 SDs. Significance was determined using the unpaired, 1-tailed Student’s *t* test. (**C**) Summary of autoantibodies in Prm5-cKO mice. Each pentagon represents an individual mouse. (**D** and **E**) Lung sections from 7- to 12-month-old control mice (*n* = 5) or Prmt5-cKO mice (*n* = 4) were stained with H&E (**D**) or CD3 and DAPI or DAPI, CD4, and CD8 (**E**). Original magnification, ×4 (**D**). Scale bar: 100 μm (**E**). Representative data from 2 independent experiments are shown. (**F**) Relative expression of melanoma antigens in mTEC^hi^ cells. (**G** and **H**) B16F10 Red-FLuc melanoma cells (5 × 10^5^) were injected into the tail vein of 6-month-old mice. Representative ex vivo bioluminescence images of the lungs 14 days after tumor injection (**G**). Quantification of the bioluminescence (arbitrary unit minus background: AU–BG) (**H**). (**I**–**K**) Flow cytometric analysis to determine CD44 and CD62L expression in left lung CD8^+^ T cells from 6-month-old control mice (*n* = 8) and Prmt5-cKO mice (*n* = 7) 14 days after tumor injection and the frequency of naive, effector, and central memory of CD8^+^ T cells (**I**). Intracellular staining for IFN-γ after PMA plus ionomycin stimulation in lung CD8^+^ T cells from the indicated mice and the frequency of lung IFN-γ^+^CD8^+^ T cells (**J**). Frequency and number of lung Tregs from the indicated mice (**K**). *P < 0.5 and **P < 0.01, by 2-tailed Student’s *t* test (**H**–**K**).

The above findings prompted us to examine whether Prmt5-cKO mice exhibit an autoimmune phenotype. Aged (7- to 12-month-old) Prmt5-cKO mice showed a mild-to-moderate production of serum autoantibodies against the kidney (5 of 8), liver (4 of 8), lung (3 of 8), salivary gland (3 of 8), and pancreas (2 of 8), whereas 1 of 6 control mice exhibited mild autoantibody production (Figure 7, A–C). We also observed massive infiltration of lymphocytes including CD4^+^ and CD8^+^ T cells into lung tissues observed in Prmt5-cKO mice (Figure 7, D and E). In these mice, splenic CD4^+^ and CD8^+^ T cells were skewed toward a CD44^+^CD62L^–^ activated phenotype (Supplemental Figure 8, A–C). Consistent with this, the splenic T cells from Prmt5-cKO mice showed increased IFN-γ production compared with those from control mice (Supplemental Figure 8D). These results indicate that Prmt5 in mTECs was required for the induction of central T cell tolerance.

The TRAs expressed by mTECs include tumor-associated antigens, the expression of which induces deletion of tumor-reactive T cells in the thymic medulla, thereby attenuating antitumor T cell immunity (35). Our RNA-Seq results showed that the expression levels of representative melanoma antigens (gp100, tyrosinase, Trp-1 and Trp-2) were reduced in mTEC^hi^ cells of Prmt5-cKO mice (Figure 7F). It is therefore possible that antitumor immunity to melanoma was enhanced in Prmt5-cKO mice. Since T cell infiltration was exaggerated in the lungs of Prmt5-cKO mice, we investigated whether TEC-specific Prmt5 deficiency promotes antitumor immunity against B16F10 melanoma using a mouse model of lung metastasis. We intravenously injected the luciferase-labeled melanoma cell line B16F10 Red-FLuc, which allows tumor burden quantification by measuring the bioluminescence level (36). Fourteen days after injection, we observed substantial lung metastasis in the control mice (Figure 7, G and H), but metastasis was markedly decreased in the Prmt5-cKO mice. The frequency of effector and central memory CD8^+^ T cell as well as IFN-γ–producing CD8^+^ T cells was significantly elevated in the lungs of Prmt5-cKO mice, along with metastasis (Figure 7, I and J). There was no change in the number or frequency of Tregs in the metastasized lung (Figure 7K). Therefore, lung metastasis was significantly suppressed by a TEC-specific loss of Prmt5, possibly because of an enhanced antitumor immunity.

## Discussion

In this study, we explored the physiological significance of arginine methylation in TECs, focusing on Prmt5, a type II arginine methyltransferase. Prmt5 is highly expressed in mature mTECs, where it catalyzes symmetric dimethylation of arginine residues in various proteins. The target proteins of Prmt5 in mTECs include SmD3, a small nuclear ribonucleoprotein whose arginine methylation is known to be involved in pre-mRNA splicing (21–23). Prmt5-deficient mTECs exhibited a marked reduction of mature mRNA and an increase of unspliced pre-mRNA, resulting in the decreased expression of various proteins. We found that Prmt5 was required for the pre-mRNA splicing and subsequent protein expression of Aire, a key factor in immune tolerance induction, and that the expression of many Aire-dependent TRAs was reduced in Prmt5-deficient mTECs. Interestingly, the expression of a large number of Aire-independent TRAs was also reduced in Prmt5-deficient mTECs due to the inhibition of pre-mRNA splicing. TEC-specific, Prmt5-deficient mice developed spontaneous autoimmune disorders in peripheral organs, accompanied by altered thymic T cell selection and an activated phenotype of peripheral T cells. These results demonstrate that Prmt5 in mTECs was essential for the induction of T cell self-tolerance, shedding light on a molecular link that runs from the pre-mRNA splicing through TRA expression to T cell repertoire selection and, ultimately, protection from autoimmunity. Further studies are needed to elucidate the significance of Prmt5 functions other than pre-mRNA splicing such as histone modification, DNA repair, and signal transduction (21).

The mechanism of diverse TRA expression in mTECs has thus far been addressed mainly in the context of modulation of chromatin accessibility and activation of superenhancers by Aire and its binding partners (37). Transcription factors such as *SpiB*, *Fezf2*, *Ascl1*, and *Sox4* have also been thought to participate in the expression of a set of TRAs (14). In the Prmt5-deficient mTECs, the expression of a large number of Aire-independent TRAs was significantly dampened without affecting the expression of these transcriptional factors. The regulation of the pre-mRNA splicing of TRAs by Prmt5 is a mechanism for maximizing the diversity of the self-antigen repertoire expressed in mTECs. This is a mechanism that acts independently of the regulation by Aire and other transcription factors, providing another layer of mTEC function. The fact that Prmt5 was expressed at the highest level in mature mTECs expressing Aire indicates the importance of pre-mRNA splicing in the production of a diverse antigen repertoire for inducing T cell tolerance in the thymus. Other PRMT members are also expressed in mTECs, albeit at a lower level than Prmt5. Their roles in mTECs are currently unclear and require further investigation. Furthermore, recent studies have demonstrated that the mTECs in the post-Aire stages undergo terminal differentiation, which allows the expression of TRAs specific for such differentiated epithelial cell types. The differentiation is either dependent on Aire (e.g., corneocyte-like mTECs) or independent of Aire (e.g., thymic tuft cells) (16, 17, 19, 38). Prmt5-deficient mTECs failed to differentiate into thymic tuft cells, indicating that Prmt5 also controls an Aire-independent differentiation program in mTECs. It is likely that the thymic medulla needs to contain a variety of cell types including terminally differentiated mTECs as well as non-TEC stromal cells such as fibroblasts in order to effectively achieve an extensive coverage of self-antigens.

It has been shown that mTECs can express alternatively spliced transcripts that are differentially expressed across multiple tissues. Recent studies demonstrated that Rbfox1/2 and Raver2, RNA-binding proteins known to be involved in alternative mRNA splicing, play a role in inducing the alternative splicing in mTECs, contributing to the broad range of coverage of self-antigen expression (39, 40). However, the significance of these factors in the induction of central tolerance has not been reportedly addressed to date. Another set of studies showed that Aire itself is responsible for the splicing control of TRAs in mTECs. The overexpression of Aire in cell lines facilitates the splicing of the reporter gene and endogenous TRA genes such as *Krt14*, and Aire-deficient mice display increased exon skipping in certain genes in mTECs (33, 41). Consistent with these observations, our results showed that the intron retention of Aire-dependent genes was increased in Aire-deficient mTECs. It was also reported that the Aire protein interacts with pre-mRNA processing factors, including SmD3, although it remains unclear how Aire controls the SmD3-containing spliceosomes. Aire does not contain the GRG motif, a preferential target amino acid sequence of Prmt5 (42), suggesting that Aire is not a direct target of Prmt5. Prmt5 has not been identified as a direct Aire binding partner, and the arginine methylation of SmD3 normally occurs in Aire-deficient mTECs, indicating that Aire-driven pre-mRNA splicing is not mediated by Prmt5-dependent arginine methylation. Prmt5 controls the pre-mRNA splicing of a number of Aire-independent TRAs in both mTEC^hi^ cells and mTEC^lo^ cells, thereby covering a wide range of types of TRA expression in the thymus, highlighting the importance of the pre-mRNA splicing by Prmt5 in achieving a diversity of TRA expression levels.

Pre-mRNA splicing, the process by which introns are removed from newly transcribed pre-mRNA, is essential for the production of protein-coding mRNAs in eukaryotes, and arginine methylation by Prmt5 is crucial for the pre-mRNA splicing of a certain set of genes in a variety of mammalian cells. The *cis*-regulatory sequences in pre-mRNA that specify Prmt5-dependent splicing have not been identified yet. It is notable that Aire is included in targets of Prmt5-mediated splicing in mTECs. A previous study has reported that Jmjd6, a bifunctional protein with arginine demethylase and lysyl hydroxylase activities, is important for the pre-mRNA splicing of Aire (43). Deficiency of Jmjd6 in mTECs resulted in an increase of the retention of intron 2 in Aire mRNA and a significant decrease in the Aire protein, although the molecular mechanism underlying how Jmjd6 controls the excision of the specific intron remains unclear. Our present data demonstrate that the splicing of most introns of the Aire gene, including intron 2, is under the control of Prmt5 via the arginine methylation of Sm proteins. Thus, it is likely that Prmt5 controls a wider range of pre-mRNA splicing in mTECs than does Jmjd6, although how these 2 factors are coordinated is still unclear. These findings on the splicing control of Aire as well as many TRA genes in mTECs will be important for future studies striving toward an understanding of the molecular basis of pre-mRNA splicing. Collectively, mature mTECs are equipped with highly ordered splicing machinery, in which Prmt5-mediated arginine methylation plays a central role, to ensure the expression of a wide variety of proteins as self-antigens. Our data showed that autoantibodies against kidney and liver are frequently detected in Prmt5-cKO mice. It will be interesting to identify the self-antigens responsible for the induction of organ-specific autoimmunity in Prmt5-cKO mice.

TRA expression by mTECs not only suppresses autoimmunity, but also dampens anti-tumor immunity by excessively removing self-reactive T cells, illustrating the double-edged potential in the immune response (35). Depletion of mTECs with an anti-RANKL antibody rescues T cells reactive to melanocyte antigens from thymic negative selection and promotes enhanced anti-melanoma T cell immunity (44). A similar phenomenon has also been observed in mice that have a hypomorphic mutation of Aire (45–47). Indeed, autoantibodies against cancer testis antigens have been found in patients with congenital AIRE deficiency (48). Thus, inhibition of mTEC development or Aire function/expression may be a potential therapeutic approach to enhance antitumor immunity. Our results show that the deletion of Prmt5 in TECs modulates the expression of self-antigens in mTECs and thereby reinforces antitumor immunity. Currently, chemical inhibitors of Prmt5 are considered to be a promising candidate for the treatment of cancer by inhibiting tumor growth, and 5 types of Prmt5 inhibitors are being tested in clinical trials (49). The present results imply that Prmt5 inhibitors may enhance T cell–mediated antitumor activity by modulating the thymic expression of self-antigens, the effect of which should be proven in the future studies. Given that Prmt5 is also important for T cell differentiation and activation, it is evidently desirable to develop drugs that modulate Prmt5 activity in a cell-type–specific manner.

In conclusion, protein arginine methylation by Prmt5 was essential for the pre-mRNA splicing and consequent protein expression of Aire and many TRAs in mTECs, maximizing the diversity of self-antigens in the thymus. The expression of self-antigens led to the elimination of self-reactive T cells and induction of central immune tolerance. Prmt5 plays a central role in arginine methylation in mTECs and is a potential therapeutic target in autoimmunity and cancer immunotherapy.

## Methods

### Sex as a biological variable.

Our study examined male and female animals, and similar findings are reported for both sexes.

### Mice.

C57BL/6N mice were purchased from SLC Japan. Prmt5^fl/fl^ mice (24), Foxn1-Cre mice (28), and Rag2-KO mice (50) were previously described. Aire-deficient mice were generated by CRISPR/Cas9-mediated genome editing, also as described previously (51). The target sequence containing the protospacer adjacent motif (PAM) (underlined) was as follows for Aire: GGTGGCAGTACCGCGGCCTTGGG. All mice were bred and maintained under specific pathogen–free conditions in our animal facility and were euthanized by an overdose of inhalational anesthetics.

### Cell preparation.

The thymus was digested into a single-cell suspension as previously described (52). Thymus tissue fragments were digested in RPMI-1640 containing 0.01% Liberase (Roche) and 0.01% DNase I (Roche) at 37°C for 10 minutes with gently pipetting. The dissociated cells in the supernatant were collected into cold PBS containing 2% FCS, 2 mM EDTA, and 0.01% NaN_3_ and kept on ice. The remaining fragments were continuously digested by adding fresh digestion medium. The digestion was repeated 3 times. The remaining thymic fragments were completely dissociated by passage through a 25 gauge needle. Finally, the digested thymic cells were collected by centrifugation and resuspended in cold PBS containing 2% FCS, 2 mM EDTA, and 0.01% NaN_3_ for further analysis. The spleen was ground with 2 glass slides and then filtered through a 100 mm nylon mesh to obtain a single-cell suspension. Lung tissue was minced into small pieces and digested with 0.125% collagenase D (Roche), 0.08% hyaluronidase (MilliporeSigma), and 0.01% DNase I (Roche) at 37°C for 30 minutes. The digested tissues were disrupted using a syringe and 18 gauge needle, and cells were passed through a 100 mm nylon mesh to remove tissue debris. The enzymatic reaction was stopped by adding cold PBS containing 2% FCS, 2mM EDTA, and 0.01% NaN_3_.

### Flow cytometry and cell sorting.

Flow cytometry and cell sorting were performed with the FACSCanto II and FACSAria III (BD Bioscience). Cells were treated with the Fc blocker (anti–mouse CD16/CD32; clone 2.4G2; TONBO Biosciences) prior to cell staining. Then, the cells were stained with a mixture of the antibodies at a final concentration of 1–2 mg/mL on ice for 30 minutes. For exclusion of dead cells, 7AAD was used. The monoclonal antibodies (all from BioLegend) used for flow cytometric analysis were as follows: anti-CD45 (30-F11), anti-EpCAM (G8.8), Ly51 (6C3), anti-CD80 (16-10A1), anti–MHC-II (M5/114.15.2), anti-Aire (5H12), anti-CD4 (GK1.5), anti-CD8 (53-5.8, anti-TCRβ (H58-597), anti-CD25 (C37), anti-Foxp3 (150D), anti-CD44 (IM7), anti-CD62L (MEL-14), anti-CD69 (H1.2F3), anti–IFN-γ (XMG1.2), and anti-Ki67 (16A8). Polyclonal anti-Dclk1 was purchased from Abcam (ab31704), anti-Vβ3 (KJ25), anti-Vβ4 (KT4), anti-Vβ5.1, 5.2 (MR9-4), anti-Vβ6 (RR4-7), anti-Vβ7 (TR310), anti-Vβ8.1, 8.2 (KJ16-133.18), anti-Vβ10b (B21.5), anti-Vβ12 (MR11-1), anti-Vβ13 (MR12-3), and anti-Vβ14 (no. 14-2) were purchased from BD Pharmingen. Biotinylated *Ulex europaeus* agglutinin 1 (UEA-1) was purchased from Vector Laboratories (B-1065-2). For intracellular staining of Foxp3, Aire, and Ki67, the Foxp3 Transcription Factor Staining Buffer Set (eBioscience) was used according to the manufacturer’s protocol. For the detection of Dclk1, IC Fixation Buffer (Thermo Fisher Scientific) was used. For cytokine production assay, cells were incubated with complete medium (53) containing PMA (2.5 ng/mL), ionomycin (1 μg/mL), and BD GolgiPlug at 37°C for 4 hours. The stimulated cells were fixed using IC Fixation Buffer (eBioscience) and then stained with antibodies.

### RT-PCR analysis.

Total RNA was extracted from isolated cells using the RNeasy kit (QIAGEN) and reverse-transcribed with SuperScript III (Invitrogen, Thermo Fisher Scientific) to obtain cDNA. qRT-PCR was performed with Realtime PCR Master Mix (TOYOBO) and a StepOne Real-Time PCR system (Life Technologies, Thermo Fisher Scientific). Results were normalized to *Actb* or *Gapdh* expression levels. For semiquantitative RT-PCR analysis of Aire and Ano9 mRNA, cDNA was amplified using a set of primers and Tks Gflex (TaKaRa). The primer sequences were as follows: *Aire* exon 10, 5′-TTCAGAGAAAACCAGGGGCCCA-3′; *Aire* exon 11, 5′-GCACAGTGTGCACACCGCAACA-3′; *Ano9* exon 12, 5′-GAAGAGATGGCCCTTGAGCTCA-3′; *Ano9* exon 14, 5′-ACAAAGCTTCAGGGCTACATATTTG-3′; *Gapdh* forward, 5′-CAACTTTGTCAAGCTCATTTCCTG-3′; *Gapdh* reverse, 5′-CCTCTCTTGCTCAGTGTCCTT-3′.

### Histological analysis.

Isolated tissues were embedded in OCT compound (Sakura Finetek), frozen, and sliced into 5 mm thick sections with a Cryostat (Leica). The sections were air-dried, fixed with neutral buffered formalin, and stained with H&E. For immunohistochemical staining, the sections were fixed with acetone or 4% formaldehyde and stained with the following antibodies: anti-CD205 (clone NLDC-145, BioLegend), anti–keratin 14 (K14, rabbit polyclonal, BioLegend), anti–keratin 5 (polyclonal, Covance), anti–keratin 8 (polyclonal, Progen), anti–keratin 10 (DE-K10, BioLegend), anti–pan-keratin (polyclonal, C-11, Abcam), anti-Dclk1 (polyclonal, Abcam), anti-Aire (5H12, eBioscience), anti-CD3 (145-2C11, BioLegend), anti-CD4 (RM4-5 or GK1.5, BioLegend), anti-CD8 (53-6.7, BioLegend), and polyclonal anti-Gnb3 (polyclonal, OSG00021W, Labome). Multicolor images were obtained with a BZ-9000 fluorescence microscope (Keyence).

### Autoantibody detection.

Serum was obtained from 7- to 12-month-old male and female mice. Autoantibodies were detected using tissue cryosections from Rag2-KO mice. Sections were air-dried, fixed with acetone, and incubated with 100-fold-diluted mouse serum for 30 minutes at 25°C. The stained sections were washed with PBS and further incubated with anti–mouse IgG Alexa Fluor 647 (Thermo Fisher Scientific) for 30 minutes at 25°C. Images were obtained with a BZ-9000 fluorescence microscope (Keyence) and analyzed with ImageJ (NIH) for the quantification of fluorescence intensity.

### RNA-Seq analysis.

cDNA was synthesized and amplified using a SMART-seq v.4 Ultra Low Input RNA Kit for Sequencing (Clontech Laboratories). Data were acquired on an Ion Proton sequencer (Thermo Fisher Scientific) and analyzed using CLC genomics Workbench v.12 and GeneSpring (Agilent Technologies). Data processing was performed using the R version 3.6.3. For calculation of the TRA diversity index, the Vegan R package (version 2.5-7) was used. For calculation of the entropy score, the Entropy R package (version 1.2.1) was used.

### Western blot analysis.

Sorted cells were lysed with RIPA Buffer (Nacalai Tesque) containing a Protease Inhibitor Cocktail (MilliporeSigma). The lysate was mixed with Trident 4× Laemmli SDS Sample Buffer containing 8% 2-mercaptoethanol and then boiled at 95°C for 5 minutes. The proteins were separated using 5%–20% SDS-polyacrylamide gel (FUJIFILM) and transferred onto a PVDF membrane. The antibodies used were as follows: anti–β-actin (A5441, MilliporeSigma), anti-PRMT5 (PRMT5–21, Santa Cruz Biotechnology), anti–dimethyl-arginine, symmetric (SYM10, MilliporeSigma), and anti-Snrpd3 (ab176182, Abcam).

### TCR sequencing.

CD4^+^CD8^–^TCRβ^+^CD25^–^CD69^lo^CD62L^hi^ thymocytes were sorted as described above, and total RNA was extracted with ISOGEN (Nippon Gene). Next-generation sequencing was performed with an unbiased TCR repertoire analysis technology developed by Repertoire Genesis. An unbiased adaptor-ligation PCR was performed as described previously (54). In brief, double-stranded cDNA was synthesized with Superscript III reverse transcriptase (Invitrogen, Thermo Fisher Scientific), ligated with a 5′ adaptor oligonucleotide, and then PCR amplified with primers specific for the adaptor and TCRβ constant regions. After the amplification of TCRβ cDNA, index (barcode) sequences were added using a Nextera XT index kit v2 setA (Illumina). Sequencing was performed with the Illumina Miseq paired-end platform (2 × 300 bp). Data processing was performed using the Repertoire Analysis software originally developed by Repertoire Genesis Inc. TCR sequences were assigned using a data set of reference sequences from the international ImMunoGeneTics information system (IMGT) database (https://www.imgt.org). Nucleotide sequences of the CDR3 region were translated into amino acid sequences. A unique sequence read (USR) was defined as a sequence read having no identity with the other sequence reads. The copy number of an identical USR was automatically counted by the RG software.

### Lung metastasis.

B16F10 Red-FLuc cells (PerkinElmer) were cultured in RPMI-1640 containing 10% FCS and penicillin-streptomycin (nacalai tesque) and maintained at 37°C in a humidified 5% CO_2_ incubator. B16F10 Red-FLuc cells (5.0 × 10^5^) were injected into the tail vein of 6-month-old mice. On day 14, the mice were euthanized, and their lungs were harvested. Bioluminescence was induced by the addition of d-luciferin potassium salt (Promega) dissolved in PBS (12.5 mg/mL) as previously described (36). Quantification of tumor burden in the lung was performed by ex vivo bioluminescence imaging using ImageQuant LAS 4000 (GE Healthcare). Regions of interest (ROIs) were drawn around the whole tissue. The signal from the ROIs was quantified at the quantum level using Multi Gauge software (Fujifilm).

### Statistics.

Data were analyzed using GraphPad Prism, version 7.0d (GraphPad Software) and R, version 3.6.3. Statistical tests, *n* values, replicate experiments, and *P* values are all presented in the figure legends. All data are expressed as the mean ± SEM. *P* values were calculated using 2-tailed Student’s *t* test or 1-way ANOVA with Dunnett’s multiple-comparison test. A *P* value of less than 0.05 was considered significant.

### Study approval.

All animal experiments were performed with the approval of the IACUC of the University of Tokyo (approval no. A2023M110-01) and the Natural Center for Global Health and Medicine’s Research Institute (approval no. 2023-A045) and were conducted in accordance with institutional guidelines.

### Data availability.

The RNA-Seq datasets generated in this study are available in the Gene Expression Omnibus (GEO) database (www.ncbi.nlm.nih.gov/geo; GEO GSE221114). The full source code are available in GitHub (https://github.com/nittatakeshi/ImmunolRev_Fig7/commit/f8d8a8098f8173af01739fd47da4128eb7894739; commit ID: f8d8a80). For all data values for all graphs, see the Supporting Data Values file.

## Author contributions

RM designed and performed most of the experiments and bioinformatics analysis. T Nitta, SN, MT, and TA performed the experiments. KN, TO, and T Nakashima generated genetically modified mice. KO provided advice on project design and data interpretation. RM, T Nitta, and HT interpreted the results and wrote the manuscript. HT supervised the project.

## Supplementary Material

Supplemental data

Unedited blot and gel images

Supporting data values

## Version 1 10/15/2024

Electronic publication
