# Supplementary material for: Transcript splicing optimizes the thymic self-antigen repertoire to suppress autoimmunity
Source: J Clin Invest. 2024 Oct 15;134(20):e179612. doi: 10.1172/JCI179612 (PMC11473167; doi:10.1172/JCI179612)
Supplement: Supplemental data [file jci-134-179612-s114.pdf]

Supplemental Material

Transcript splicing optimizes the thymic self-antigen repertoire to suppress autoimmunity

Ryunosuke Muro[1,2], Takeshi Nitta[1,2], Sachiko Nitta[1], Masayuki Tsukasaki[3], Tatsuo Asano[3], Kenta Nakano[4], Tadashi Okamura[4], Tomoki Nakashima[5], Kazuo Okamoto[3,6], Hiroshi Takayanagi[1]*

[1]Department of Immunology, Graduate School of Medicine and Faculty of Medicine, The University of Tokyo, Tokyo, Japan

[2]Division of Molecular Pathology, Research Institute for Biomedical Sciences, Tokyo University of Science, Chiba, Japan

[3]Department of Osteoimmunology, Graduate School of Medicine and Faculty of Medicine, The University of Tokyo, Tokyo, Japan.

4Department of Laboratory Animal Medicine, Research Institute, National Center for Global Health and Medicine, Tokyo, Japan.

[5]Department of Cell Signaling, Graduate School of Medical and Dental Sciences, Tokyo Medical and Dental University, Tokyo, Japan.

[6]Division of Immune Environment Dynamics, Cancer Research Institute, Kanazawa University, Kanazawa, Japan

*** Correspondence:**

Address: 7-3-1, Hongo Bunkyo-ku, Tokyo 113-0033

Tel: +81-3-5841-3373   Fax: +81-3-5841-3450   Email: takayana@m.u-tokyo.ac.jp

Supplemental Figure 1

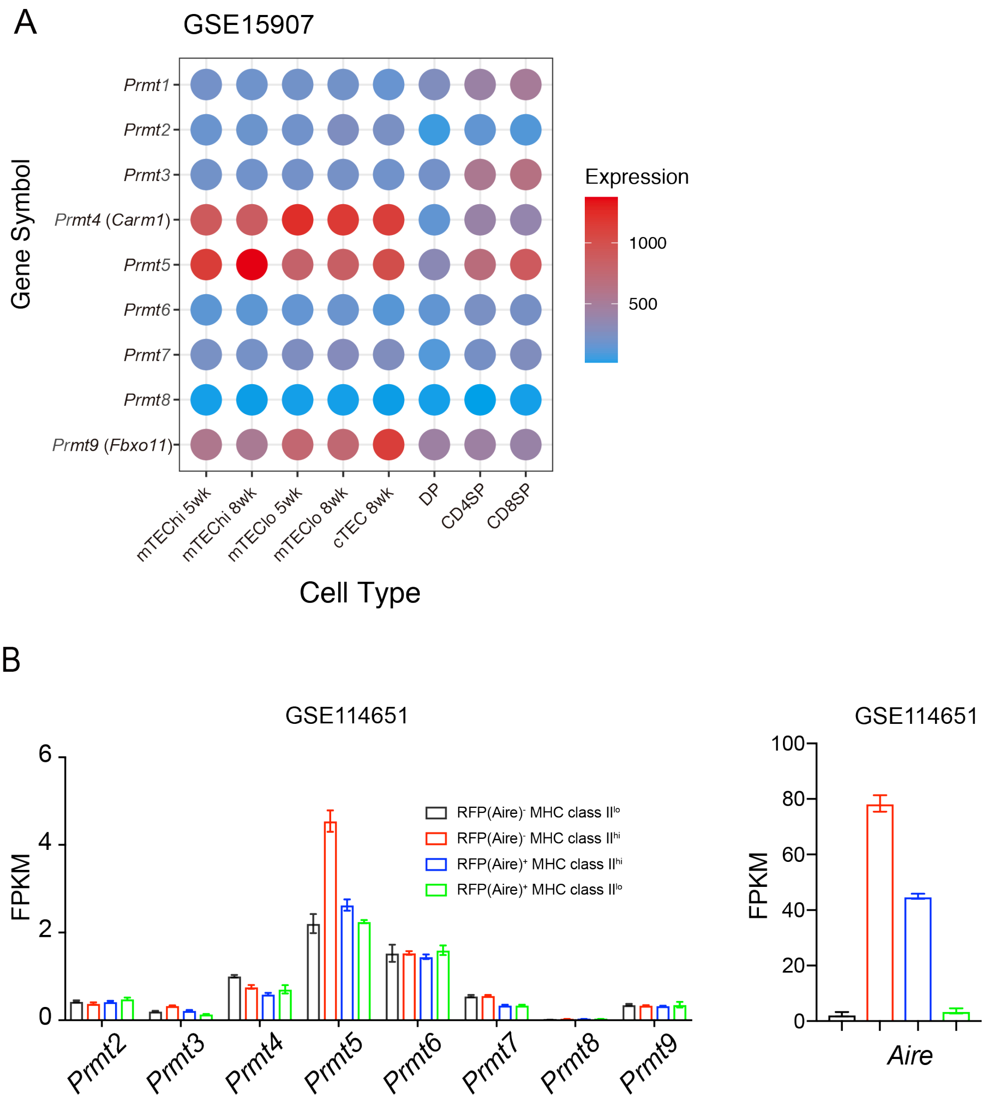

Supplemental Figure 1. Expression of PRMT family genes in thymic epithelial cells. **(A)** Expression of mRNA encoding each PRMT genes in isolated mouse thymic epithelial cell subsets and thymocyte subsets analyzed by DNA microarray is shown as a heatmap. The data were obtained from the Gene Expression Omnibus (GEO, GSE15907).

(B) Expression of each PRMT and Aire in mTEC subsets isolated from Aire^{creERT2} Rosa26$^{CAG\text{-}stopflox\text{-}tdTomato}$ mice after 7 days of administration of tamoxifen, analyzed by RNA-seq (17). The data was obtained from GEO (GSE114651).

Supplemental Figure 2

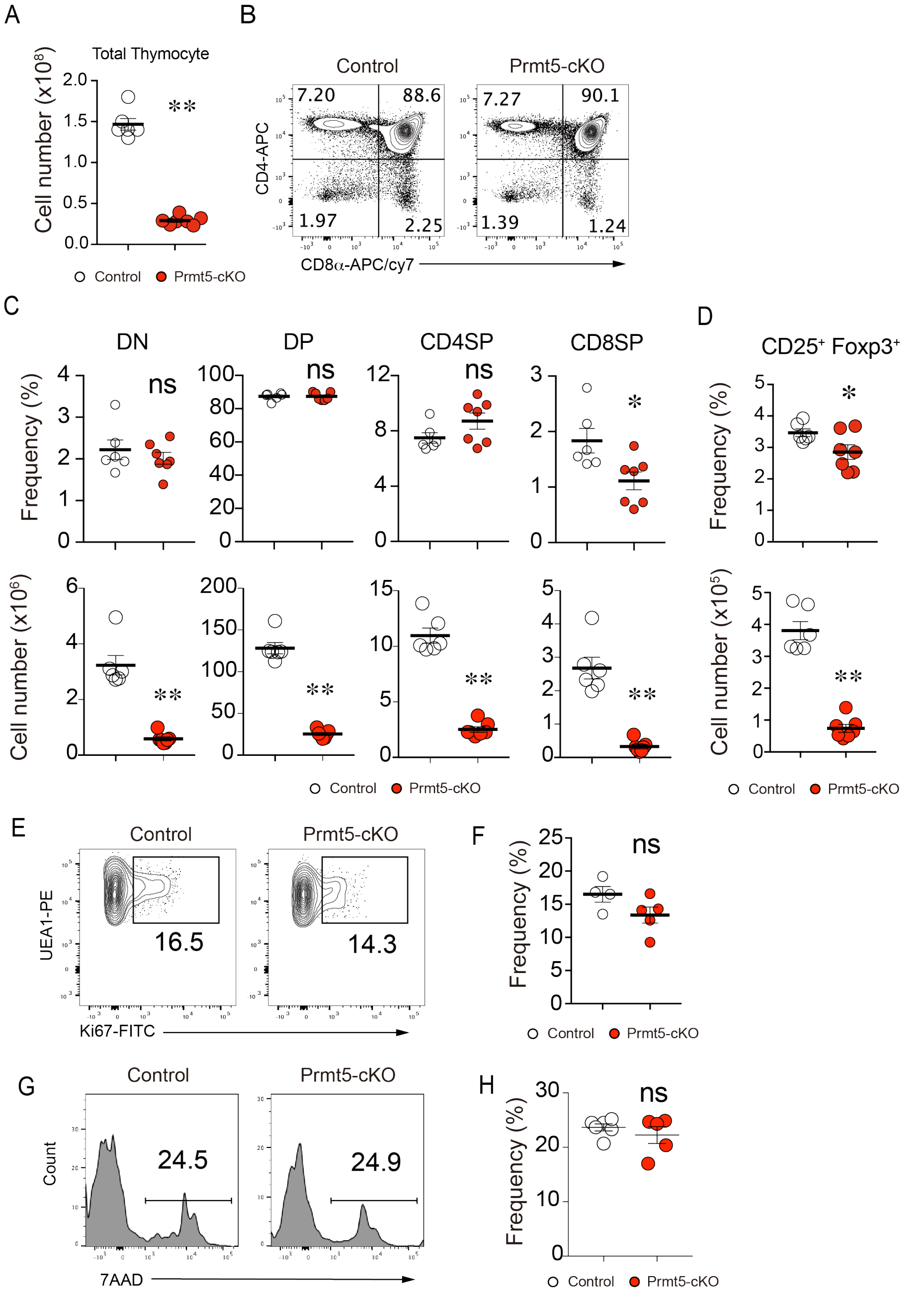

Supplemental Figure 2. Thymocyte development and mTEC proliferation in Prmt5-cKO mice.

(A) The number of thymocytes per thymus lobe in 4- to 5-week-old control mice ($Prmt5^{flox/flox}$, n = 6) and Prmt5-cKO mice ($Prmt5^{flox/flox}$ Foxn1-Cre, n = 7). Each circle indicates one mouse.

(B and C) Representative flow cytometric profiles for CD4 and CD8 expression in thymocytes from the indicated mice (**B**) and the frequency (upper) and cell number (lower) of DN ($CD4^{–} CD8^{–}$), DP ($CD4^{+} CD8^{+}$), CD4SP ($CD4^{+} CD8^{–} TCR\beta^{+}$) and CD8SP ($CD4^{+} CD8^{–} TCR\beta^{+}$) cells (**C**).

(D) The frequency (upper) and cell number (lower) of thymic Treg cells ($CD4^{+} CD8^{–} TCR\beta^{+} CD25^{+} Foxp3^{+}$) in the indicated mice.

(E and F) Flow cytometric analysis of Ki67-expressing mTECs ($CD45^{–}$ $EpCAM^{+}$ UEA-1^{+}) from control mice (n = 4) and Prmt5-cKO mice (n = 5) (**E**). The graph depicts the frequency of $Ki67^{+}$ mTECs (**F**).

(G and **H)** Flow cytometric analysis of 7-aminoactinomycin D (7AAD) positive mTEC from control mice (n = 5) and Prmt5-cKO mice (n = 6) **(G)**. The graph depicts the frequency of $7AAD^{+}$ mTECs (**H**).

In (**A**), (**C** and **D**), (**F**) and (**H**), significance was determined using the unpaired, two-tailed Student's t-test.

Supplemental Figure 3

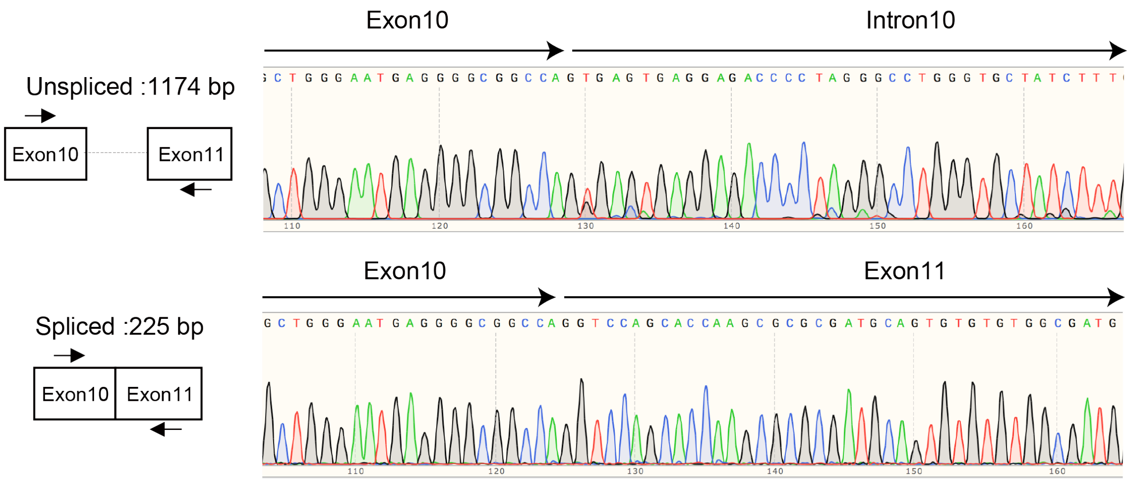

Supplemental Figure 3. Impaired splicing of Aire pre-mRNA in Prmt5-cKO mice. PCR products amplified with a set of primes spanning Aire exons 10 and 11 (unspliced: 1174 bp, spliced: 225 bp, as shown in Figure 2, E and F) were extracted from the agarose gel and sequenced by the Sanger method.

Supplemental Figure 4

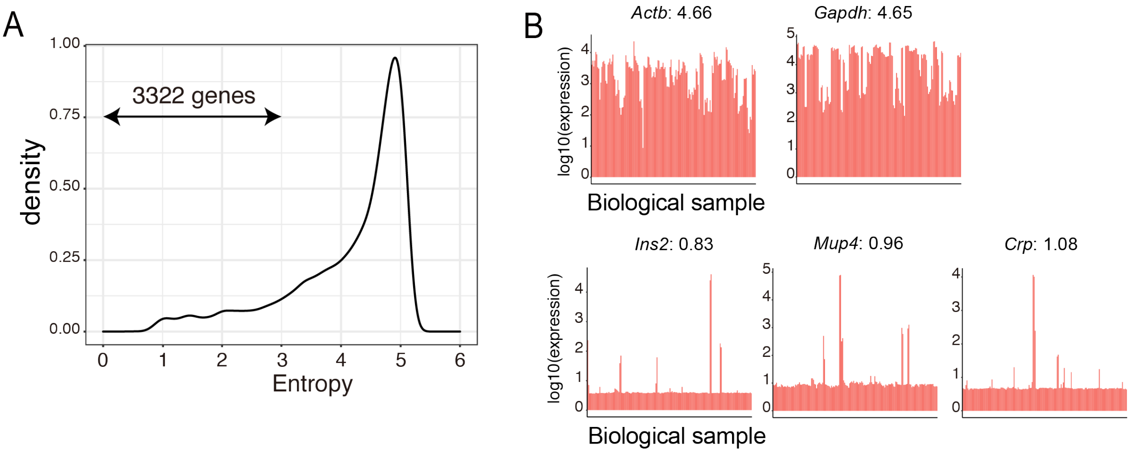

C

TRA type	Entropy score		
	< 3.0	< 3.2	< 3.5
TRA^{Prmt5}	168 (5.9%)	196 (5.7%)	252 (5.6%)
TRA^{Aire}	576 (20.1%)	689 (20.1%)	880 (19.6%)
TRA^{Shared}	651 (22.7%)	756 (22.1%)	902 (20.1%)
TRA^{other}	1471 (51.3%)	1785 (52.1%)	2458 (54.7%)
Total	2866 (100%)	3426 (100%)	4492 (100%)

Supplemental Figure 4. Definition of TRAs by the Shannon entropy score.

(A) Density plot of the entropy score for all genes. Genes with entropy score less than 3.0 were defined as TRAs.

(B) Expression profile of representative housekeeping genes (Actb and Gapdh) and TRA genes (Ins2, Mup4 and Crp) in peripheral tissues and cells (GSE10246). The number indicates the entropy score for each gene. The R codes for the calculation of the entropy score were deposited in Github (https://github.com/nittatakeshi/).

(C) The number and proportion of the indicated TRAs at the different thresholds of the entropy score.

Supplemental Figure 5

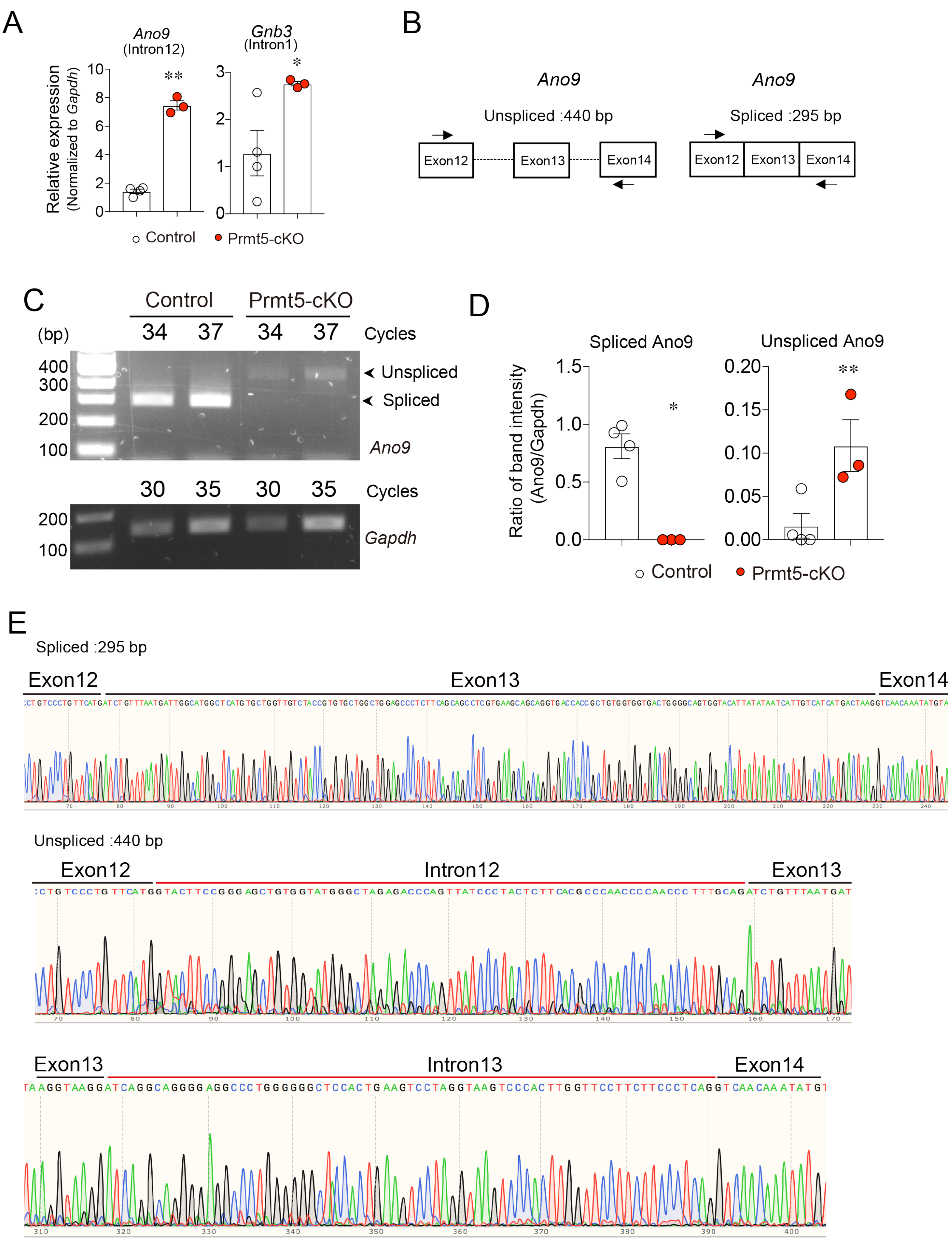

Supplemental Figure 5. Prmt5 deficiency increases the intron retention of pre-mRNAs encoding TRAs.

(A) The relative expressions of Ano9 intron 12 and Gnb3 intron 1 in mTEC^{hi} cells from

control (Prmt5$^{flox/flox}$, n = 4) and Prmt5-cKO (Prmt5$^{flox/flox}$ Foxn1-Cre, n = 3) mice were determined by qRT-PCR.

(B) Schematic of unspliced and spliced Ano9 mRNA is shown. The primers used in (**C**) are indicated by arrows.

(C) Semi-quantitative RT-PCR analysis of unspliced and spliced Ano9 mRNA in mTEC^{hi} cells from control and Prmt5-cKO mice. *Gapdh* was used as an internal control.

(D) Ratio of the band intensity of unspliced (440 bp) and spliced (295 bp) Ano9 mRNA. The band intensity was measured with Image-J, and was normalized to *Gapdh*.

(E) Nucleotide sequence of PCR products amplified with a set of primers spanning exon 12 and 14 of Ano9.

In (**A**) and (**D**), significance was determined using the unpaired, two-tailed Student's t-test.

Supplemental Figure 6

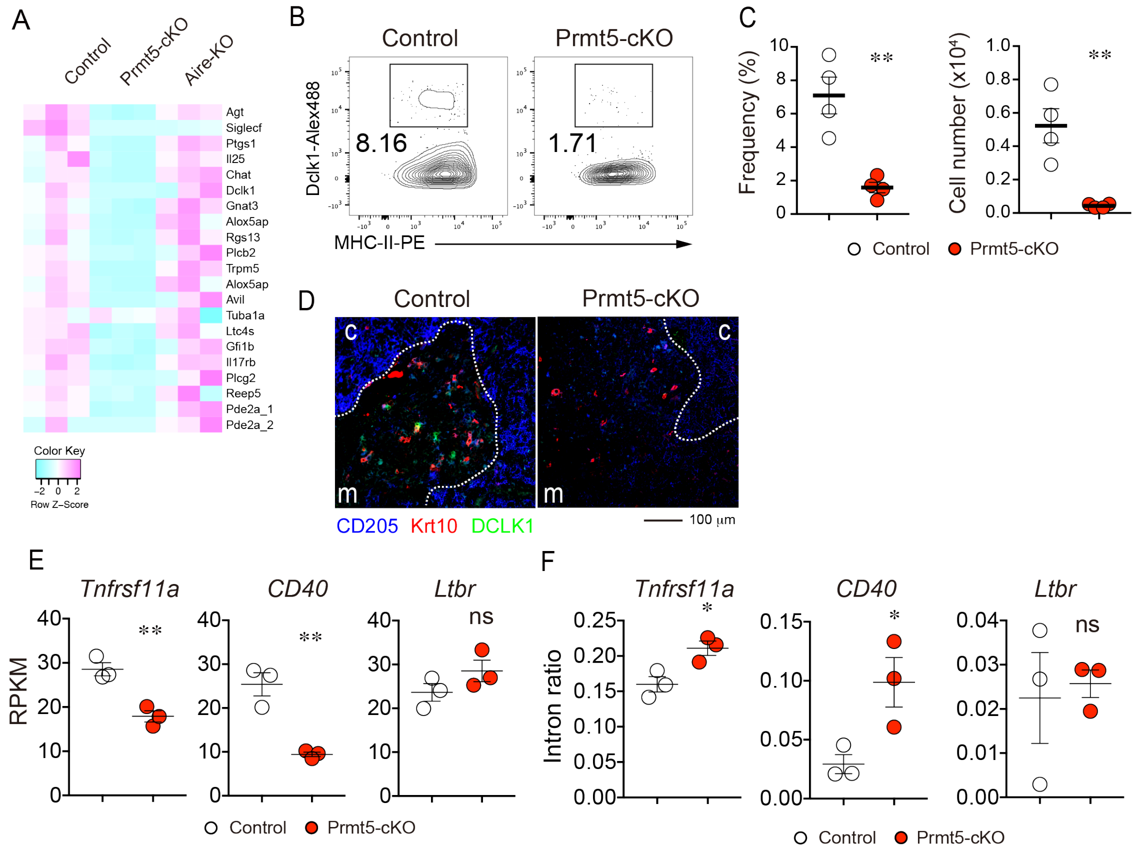

Supplemental Figure 6. Prmt5 regulates differentiation of mTECs.

(A) Heatmap showing the expression of tuft cell signature genes in the mTEC^{lo} cells from control mice (Prmt5$^{flox/flox}$, n = 3), Prmt5-cKO mice (Prmt5$^{flox/flox}$ Foxn1-Cre, n = 3) and Aire-KO mice (n = 3).

(B and C) Flow cytometric analysis of Dclk1 expression in mTEC^{lo} cells (EpCAM$^+$, UEA-1$^+$, MHC-II^{lo}) (**B**). The graphs depict the frequency and number of Dclk1$^+$ thymic tuft cells (**C**).

(D) Thymic sections from 4-week-old mice were stained with anti-CD205, anti-keratin 10 and Dclk1. The scale bar indicates 100 μm. c, cortex; m, medulla.

(E and **F)** The mRNA expression (**E**) and intron retention ratio **(F)** of *Tnfrsf11a*, *CD40* and *Ltbr* in sorted mTEC^{lo} cells.

In (**C**), **(E)** and (**F)**, significance was determined using the unpaired, two-tailed Student's t-test.

Supplemental Figure 7

A

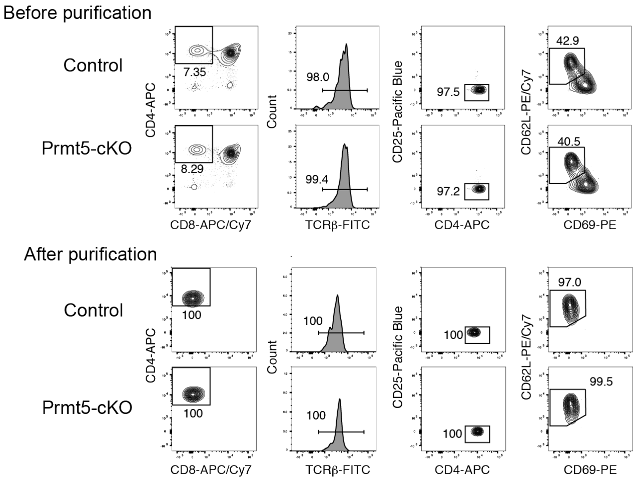

B

	CD4SP number	Total TCR reads	Unique TCR reads
Control-1	1.5×10^5	2.88×10^5	2.19×10^4
Control-2	1.5×10^5	3.63×10^5	2.67×10^4
Control-3	1.5×10^5	2.92×10^5	2.17×10^4
Mean		3.14×10^5	2.35×10^4
SE		2.43×10^4	1.62×10^3
Prmt5-1	1.5×10^5	2.91×10^5	2.05×10^4
Prmt5-2	1.5×10^5	2.50×10^5	1.81×10^4
Prmt5-3	1.5×10^5	2.50×10^5	1.98×10^4
Mean		2.64×10^5	1.94×10^4
SE		1.36×10^4	0.71×10^3

C

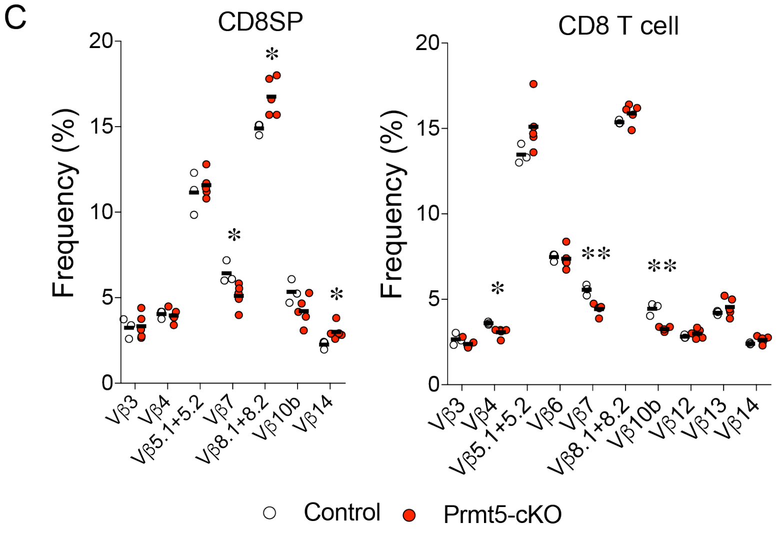

Supplemental Figure 7. TCR repertoire analysis of TEC-specific Prmt5-deficient mice.

(A) Gating strategy for the isolation of mature CD4SP cells ($CD4^+$ $CD8^-$ $TCR\beta^+$ $CD25^-$ $CD69^{lo}$ $CD62L^{hi}$).

(B) Summary of TCR repertoire sequencing. 1.5×10^5 mature CD4SP cells isolated from Control ($Prmt5^{flox/flox}$, n = 3) or Prmt5-cKO mice ($Prmt5^{flox/flox}$ Foxn1-Cre, n = 3) were used for TCR sequencing. The number of total and unique TCR reads is shown.

(C) Usage of TCR–Vβ in CD8SP thymocyte and splenic CD8 T cell from control mice (n = 3) and Prmt5-cKO mice (n = 5) were analyzed with flow cytometry. The significance was determined using the unpaired, two-tailed Student's t-test.

Supplemental Figure 8

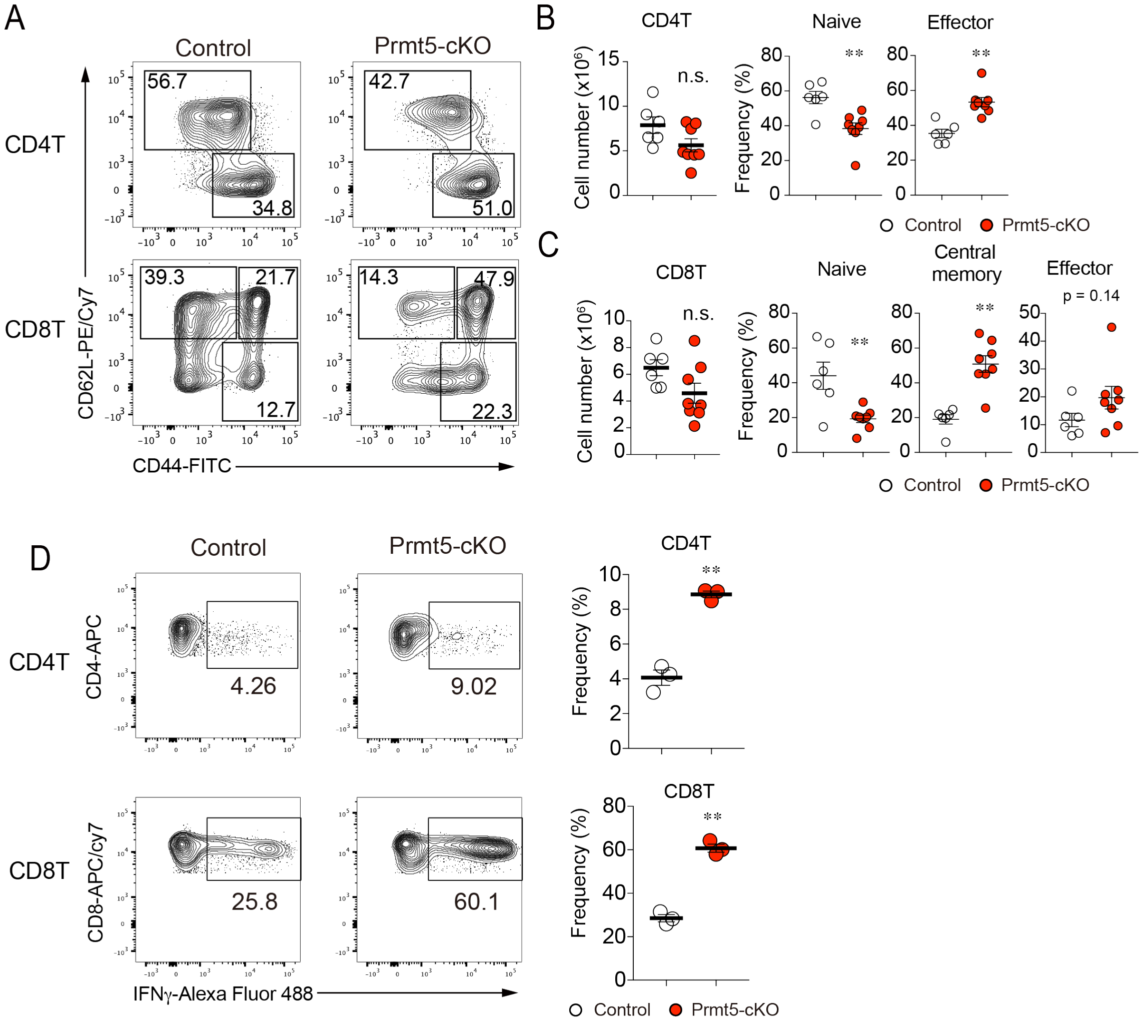

Supplemental Figure 8. Analysis of peripheral T cells in aged TEC-specific Prmt5-deficient mice.

(A) Flow cytometric analysis of CD44 and CD62L expression in splenic CD4 T cells and CD8 T cells from 7- to 12-month-old control mice ($Prmt5^{flox/flox}$, n = 6) and Prmt5-cKO mice ($Prmt5^{flox/flox}$ Foxn1-Cre, n = 8).

(B) The numbers of total CD4 T cells ($CD4^{+}$ $CD8^{-}$ $TCR\beta^{+}$), and the frequency of their subpopulations (naïve: $CD44^{-}$ $CD62L^{+}$, effector: $CD44^{+}$ $CD62L^{+}$).

(C) The numbers of total CD8 T cells ($CD4^{-}$ $CD8^{+}$ $TCR\beta^{+}$) and the frequency of their subpopulations (naïve: $CD44^{-}$ $CD62L^{+}$, effector: $CD44^{+}$ $CD62L^{-}$, central memory:

CD44^{+} CD62L^{+}).

(D) Intracellular staining for IFNγ after stimulation with PMA and ionomycin in splenic CD4 or CD8 T cells from 10-month-old control mice (Prmt5$^{flox/flox}$, n = 3) and Prmt5-cKO mice (Prmt5$^{flox/flox}$ Foxn1-Cre, n = 3).

In **(B)**, (**C)** and **(D)** significance was determined using the unpaired, two-tailed Student's t-test.
