# Supplementary material for: Transcript splicing optimizes the thymic self-antigen repertoire to suppress autoimmunity
Source: J Clin Invest. 2024 Oct 15;134(20):e179612. doi: 10.1172/JCI179612 (PMC11473167; doi:10.1172/JCI179612)
Supplement: Unedited blot and gel images [file jci-134-179612-s115.pdf]

Full unedited gels for Figure 1I

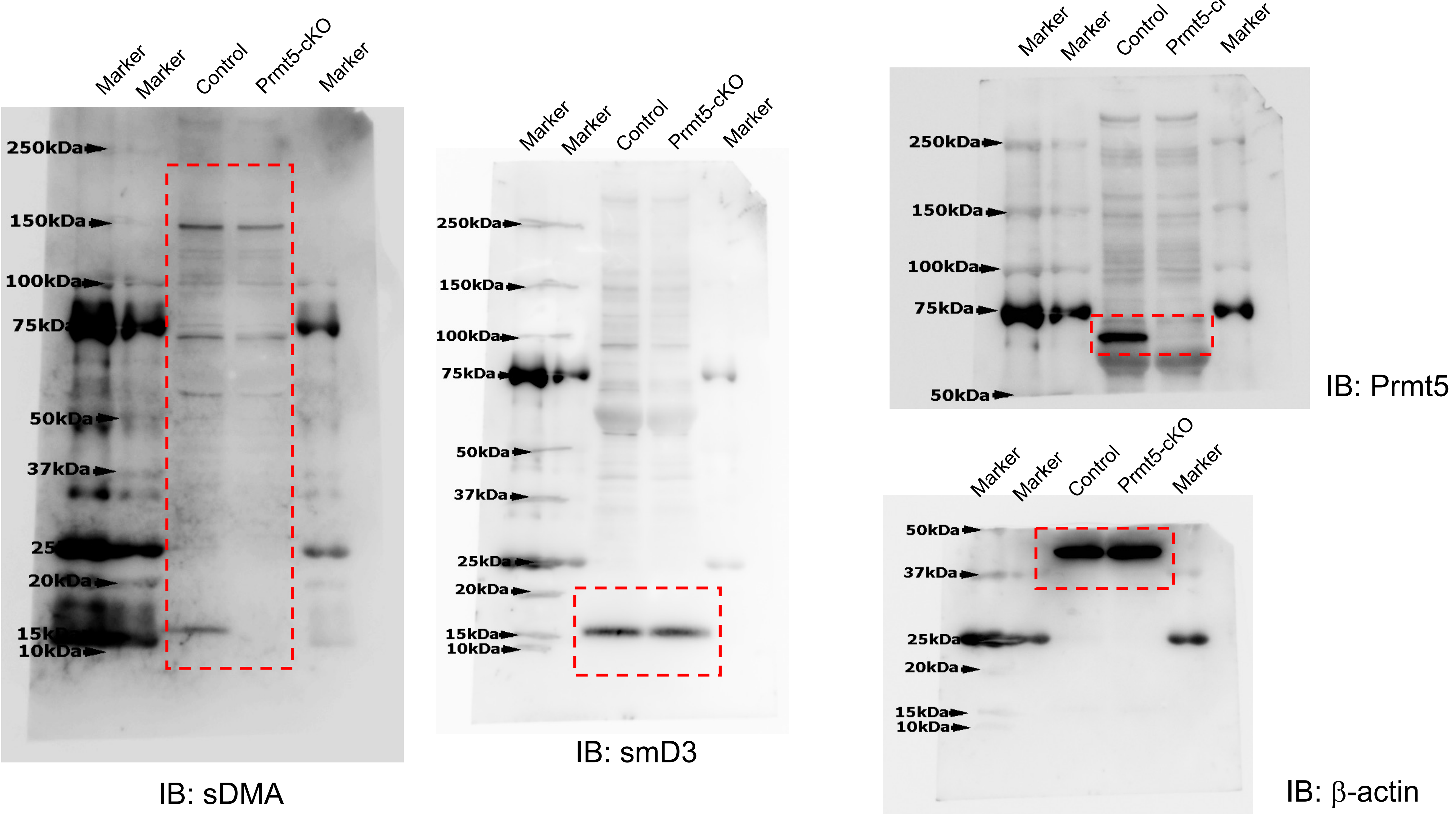

Full unedited gels for Figure 4F

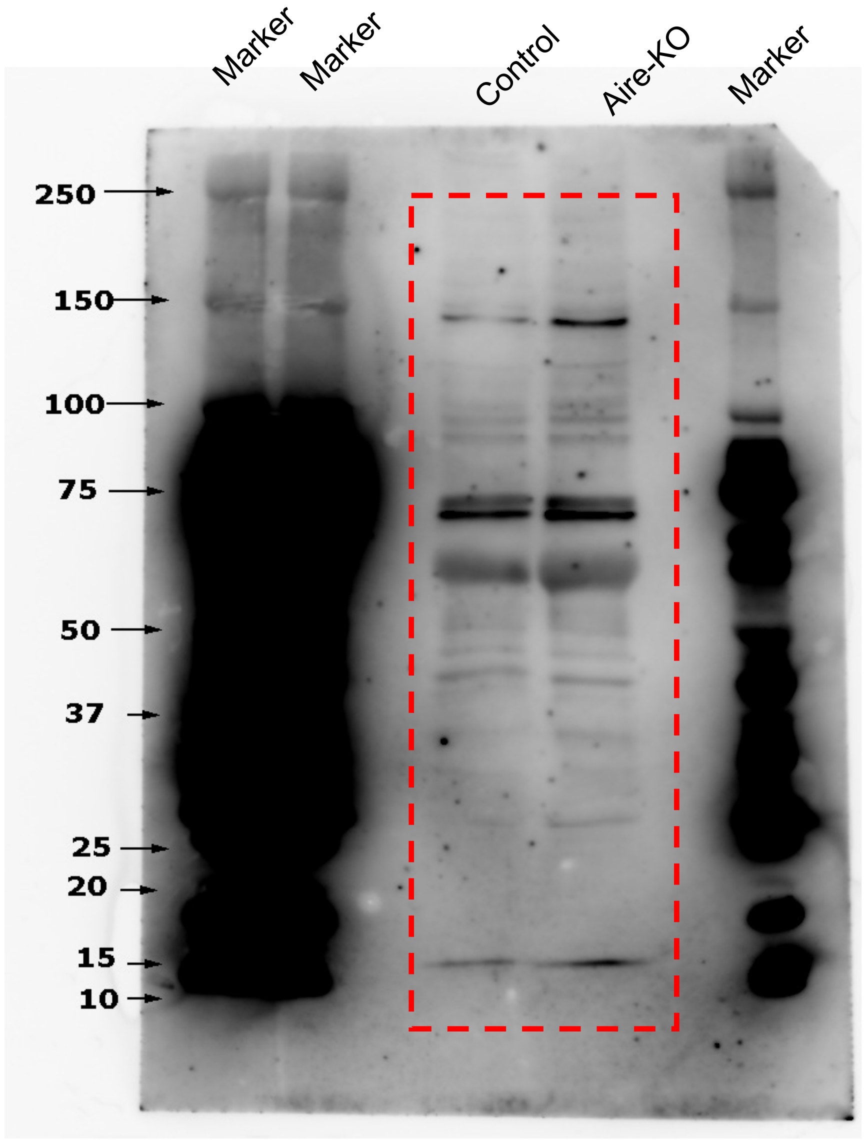

IB: sDMA

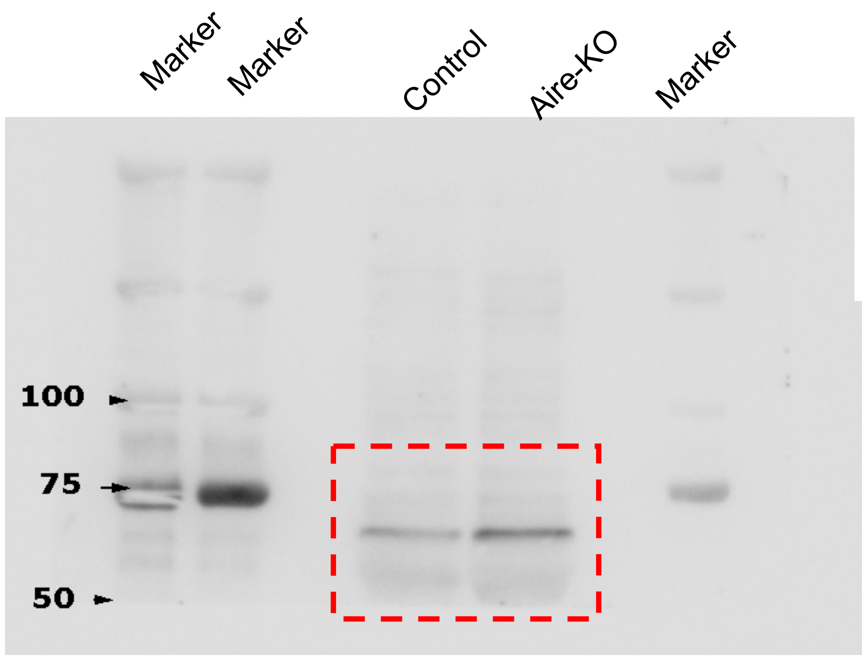

IB: Prmt5

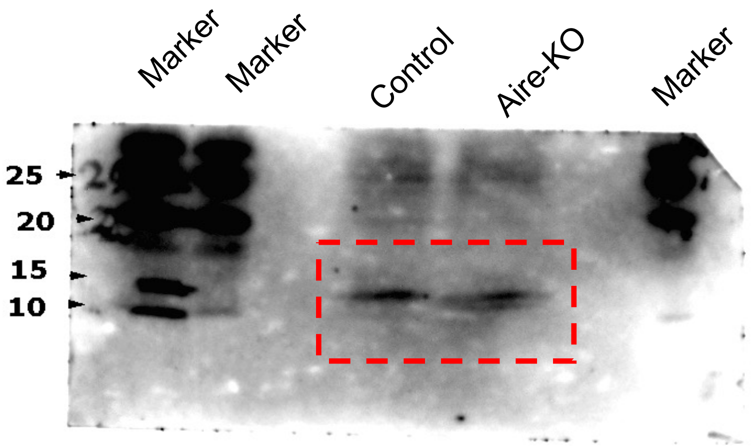

IB: SmD3

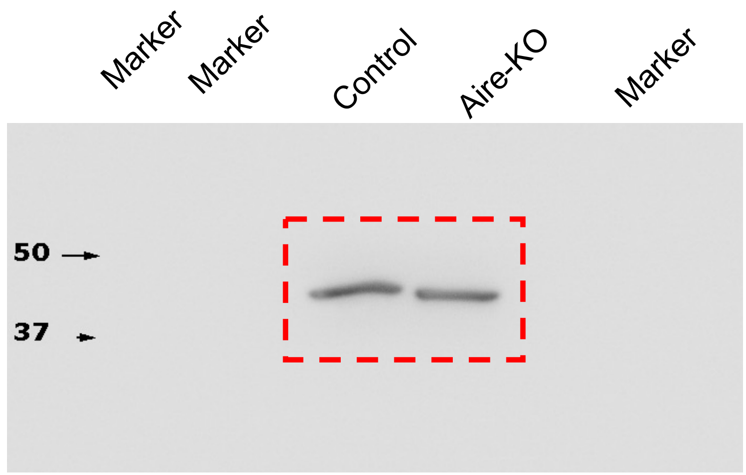

IB: β-actin

Full unedited gels for Figure 2F

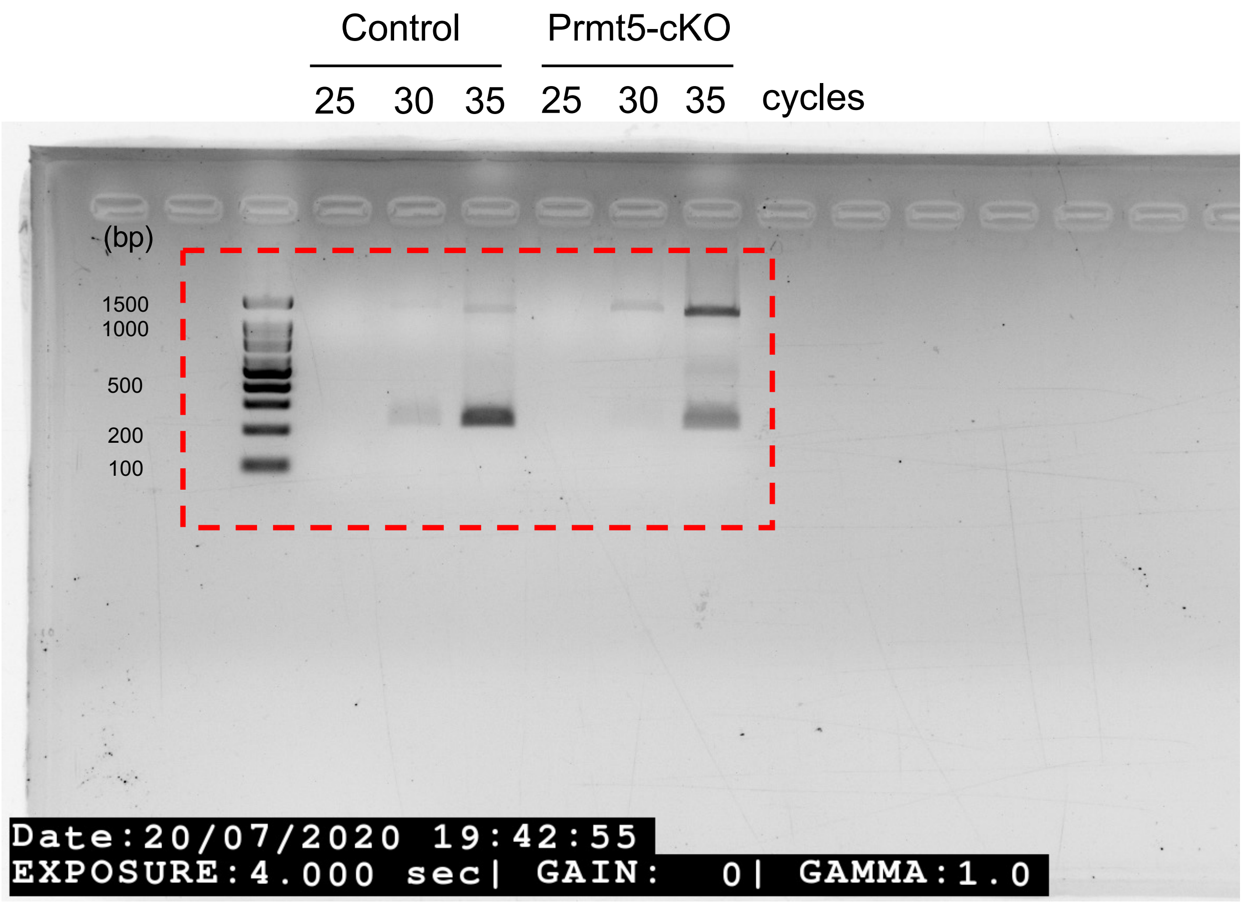

Aire

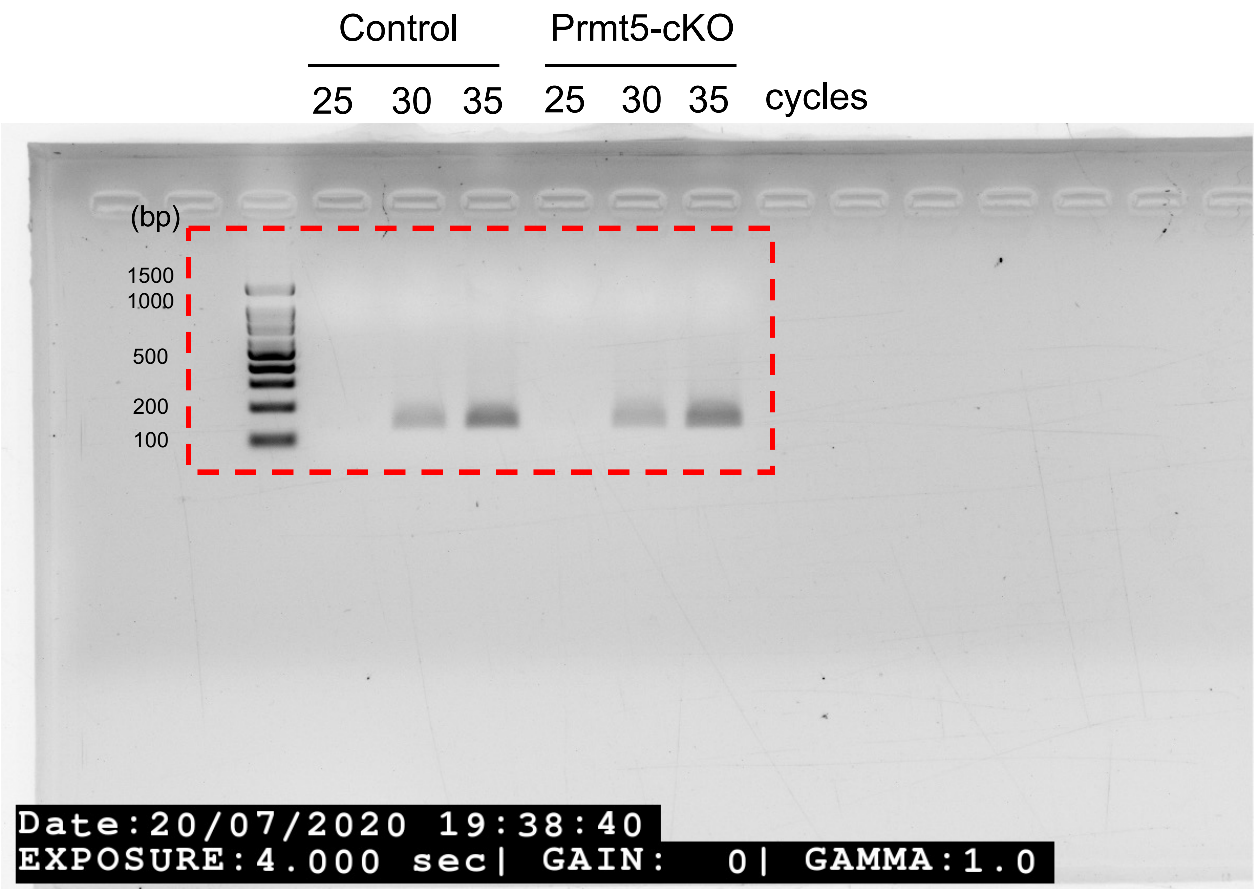

Gapdh

Full unedited gels for Supplemental Figure 5C

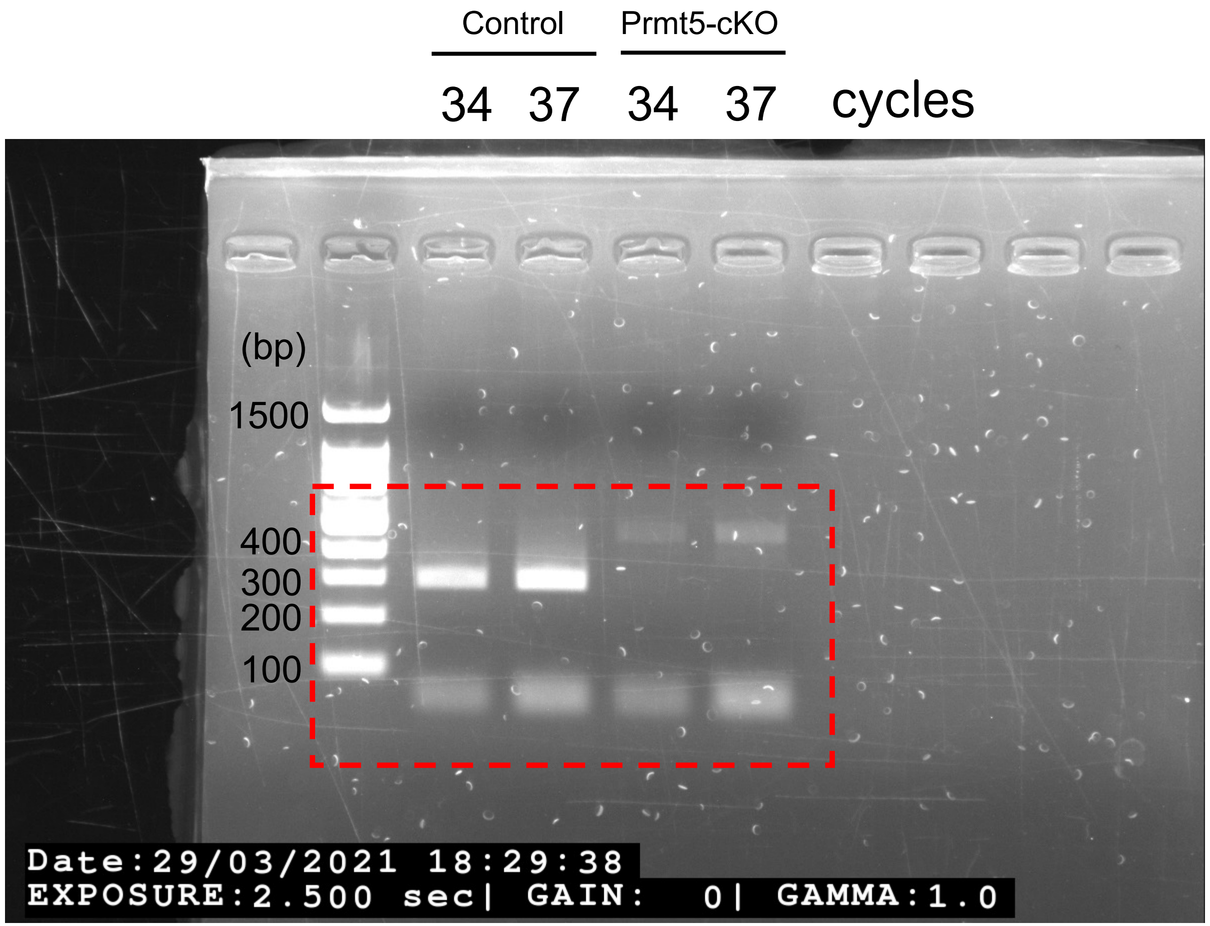

Ano9

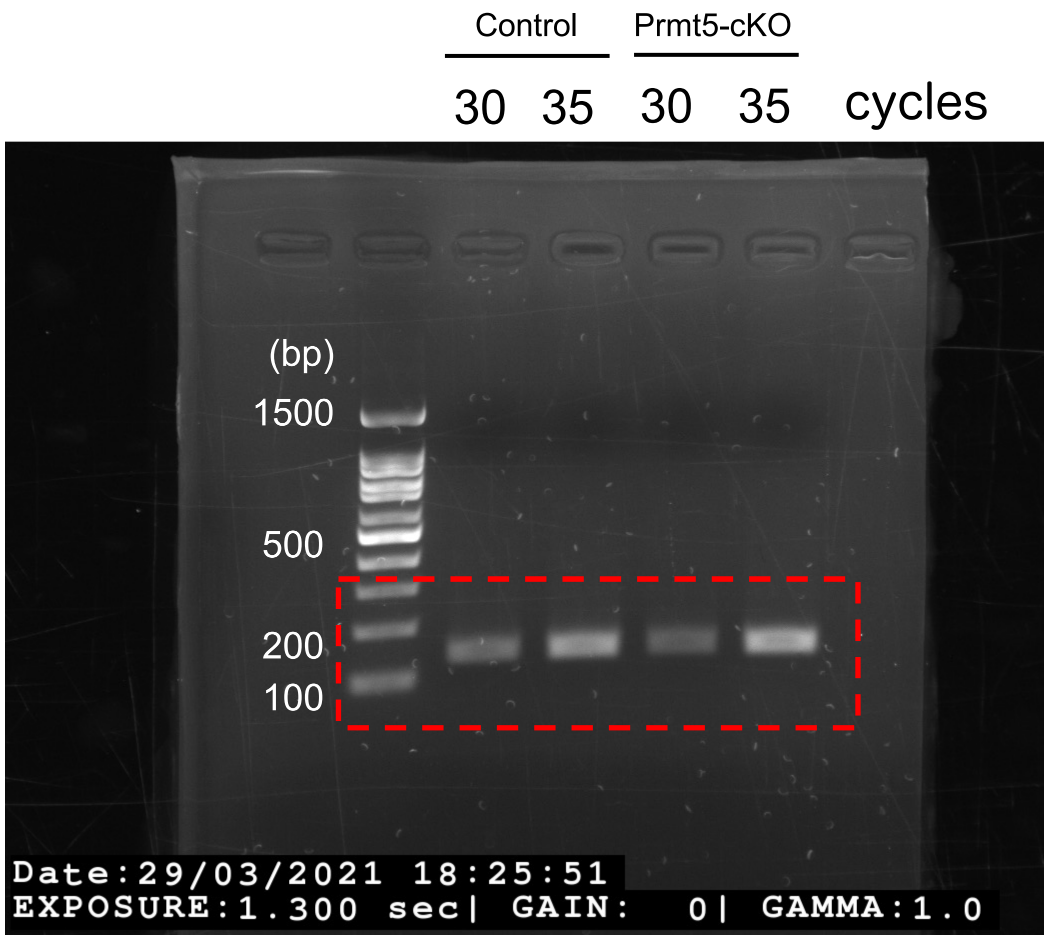

Gapdh
